# Identification of early liver toxicity gene biomarkers using comparative supervised machine learning

**DOI:** 10.1038/s41598-020-76129-8

**Published:** 2020-11-05

**Authors:** Brandi Patrice Smith, Loretta Sue Auvil, Michael Welge, Colleen Bannon Bushell, Rohit Bhargava, Navin Elango, Kamin Johnson, Zeynep Madak-Erdogan

**Affiliations:** 1 Department of Food Science and Human Nutrition, University of Illinois, 1201 W Gregory Dr, Urbana-ChampaignUrbana, IL 61801 USA; 2 Illinois Informatics Institute, University of Illinois, Urbana-Champaign, Urbana, IL USA; 3 National Center for Supercomputing Applications, University of Illinois, Urbana-Champaign, Urbana, IL USA; 4 Carl R. Woese Institute for Genomic Biology, University of Illinois, Urbana-Champaign, Urbana, IL USA; 5 Carle Illinois College of Medicine, University of Illinois, Urbana-Champaign, Urbana, IL USA; 6 Department of Bioengineering, University of Illinois, Urbana-Champaign, Urbana, IL USA; 7 Corteva Agrisciences, The Agriculture Division of DowDupont, Indianapolis, IN USA; 8 Cancer Center at Illinois, University of Illinois, Urbana-Champaign, Urbana, IL USA; 9 Beckman Institute for Advanced Science and Technology, University of Illinois at Urbana-Champaign, Urbana, IL USA

**Keywords:** Computational biology and bioinformatics, Machine learning

## Abstract

Screening agrochemicals and pharmaceuticals for potential liver toxicity is required for regulatory approval and is an expensive and time-consuming process. The identification and utilization of early exposure gene signatures and robust predictive models in regulatory toxicity testing has the potential to reduce time and costs substantially. In this study, comparative supervised machine learning approaches were applied to the rat liver TG-GATEs dataset to develop feature selection and predictive testing. We identified ten gene biomarkers using three different feature selection methods that predicted liver necrosis with high specificity and selectivity in an independent validation dataset from the Microarray Quality Control (MAQC)-II study. Nine of the ten genes that were selected with the supervised methods are involved in metabolism and detoxification (Car3, Crat, Cyp39a1, Dcd, Lbp, Scly, Slc23a1, and Tkfc) and transcriptional regulation (Ablim3). Several of these genes are also implicated in liver carcinogenesis, including Crat, Car3 and Slc23a1. Our biomarker gene signature provides high statistical accuracy and a manageable number of genes to study as indicators to potentially accelerate toxicity testing based on their ability to induce liver necrosis and, eventually, liver cancer.

## Introduction

Pathological and biochemical data in non-human mammals are used extensively by the agrochemical and pharmaceutical sectors for assessing mammalian toxicity and effects on human health of molecular innovations. This effort is extensive; in addition to other cost and effort, required mammalian toxicity assessment packages can use ~ 6000 animals per molecule studied. Despite such careful screening, major setbacks to pharmaceutical product development pipelines still result where human toxicity is detected during late stages. When toxicity is not determined in this testing, a danger to public health arises if adverse effects on humans are only observed in the population after years of deployment. These risks can be greatly mitigated if early biomarkers of eventual toxicity can be found. Toxicogenomics, or the application of genomics methods to predict adverse effects of exogenous molecule exposure^1^, is gaining popularity with advances in computing and availability of curated data sets. Toxicogenomics databases have been designed and, through rigorous experiments on rat and human cell models, provide an avenue to understand the molecular basis of adverse conditions due to chemical toxicant exposures. Computational methods provide an opportunity to develop this much-desired capability^2^. These methods are relatively low cost to develop and test, can expedite data analysis, can reduce cost by reducing the scale of animal studies, and can reduce time to market for a safe product.

Toxicogenomics analyses are commonly categorized in the big data paradigm because of the large number of gene profiles that arise from the small number of samples, thus the need for data reduction tools. Classical statistical methods of identifying differentially expressed genes from microarray or RNA sequencing data results in lists comprising thousands of genes, which is not ideal for laboratory testing. Machine learning approaches such as feature selection and classification often use robust statistical modeling to reduce the number of features or variables used in the models^3–6^. Feature selection and classification can both be achieved by supervised methods for classification or unsupervised learning methods ^5^ that are primarily used for discovery.

Studies have shown that the use of supervised classification predictive models can help to find discriminative gene signatures across multiple platforms of microarray data^3–6^. Previously, several studies have used machine learning methods for prediction of biological end points^7–9^. Despite many attempts in the field, however, predictive ability remains relatively poor due to systematic noise associated with design of gene expression experiments^10^, high number of features in the signature, low predictive performance^11–14^, or poor performance of identified biomarkers at validation stage^15^. Innovations in data analysis pipeline design and modeling are still sorely needed.

The goal of this study was to construct a suitable modeling framework based on machine learning for feature selection, feature ranking, and predictive analysis applicable to liver toxicity. The developed framework was applied to the TG-GATES data set to select and rank the gene expression features that can serve as biomarkers for liver toxicity in rats^16^. After determining these features, a set of predictive models were optimized. Finally, the model was applied to untrained MAQC-II data to evaluate liver toxicity predictions^17,18^. The targeted conclusion of our study was to determine a small set of genes that successfully predicted liver necrosis and could be used for predictive testing in animals.

## Methods

### Data sets

Gene expression data were obtained from TG-GATES database for male rat, in vivo experimental models utilizing Affymetrix Microarray Chip from the TG-GATES database https://dbarchive.biosciencedbc.jp/en/open-tggates/data-2.html. The in vivo models were categorized by whole organism outcomes of exposure related to cellular injury^19,20^. The treatments included 42 chemical compounds (Table 1, Supplementary Fig. 1A) at control, low, middle, and high dose levels and 8 time points, single dose: 3 h, 6 h, 9 h and 24 h; and repeat dose: 4 days, 8 days, 15 days and 29 days. In the single dose experiments, groups of 20 animals were administered a compound and then five animals per time point were sacrificed (3, 6, 9 or 24 h) after administration (Supplementary Fig. 1B ^16^). Livers were harvested after indicated time points. RNA was isolated, and gene expression patterns were analyzed using the common array platform, Affymetrix Rat 230 2.0 microarray that contained probes for 31,099 genes.

**Table 1:** Compounds from TG-GATES database that result in necrosis.

Compound name	Abbreviation
Acarbose	ACA
Acetamidofluorene	AAF
Acetaminophen	APAP
Ajmaline	AJM
Allopurinol	APL
Allyl alcohol	AA
Amiodarone	AM
Aspirin	ASA
Azathioprine	AZP
Captopril	CAP
Ciprofloxacin	CPX
Clofibrate	CFB
Colchicine	COL
Diclofenac	DFNa
Enalapril	ENA
Ethanol	ETN
Ethionamide	ETH
Ethionine	ET
Etoposide	ETP
Fluphenazine	FP
Furosemide	FUR
Gemfibrozil	GFZ
Griseofulvin	GF
Indomethacin	IM
Lomustine	LS
Lornoxicam	LNX
Mefenamic acid	MEF
Meloxicam	MLX
Metformin	MFM
Methyldopa	MDP
Naphthyl isothiocyanate	ANIT
Naproxen	NPX
Nitrofurantoin	NFT
Nitrofurazone	NFZ
Nitrosodiethylamine	DEN
Pemoline	PML
Ranitidine	RAN
Simvastatin	SST
Tannic acid	TAN
Tetracycline	TC
Valproic acid	VPA
WY-14643	WY

Data from the Microarray Quality Control Project (MAQC II) was used for validation and assessing classification performance of the top selected features^17^. From the six datasets, we focused on the National Institute of Environmental Health Sciences (NIEHS) data set for validation since it pertains to toxic effect of chemicals on liver. The study was similar to TG-GATES, which used microarray gene expression data acquired from the liver of rats exposed to various hepatotoxicants. Gene expression data, collected from 418 rats exposed to one of eight compounds (1, 2-dichlorobenzene, 1, 4-dichlorobenzene, bromobenzene, monocrotaline, N-nitrosomorpholine, thioacetamide, galactosamine, and diquat dibromide), were used to build classifiers for prediction of liver necrosis. Each of the eight compounds were studied and analyzed using the common array platform (Affymetrix Rat 230 2.0 microarray), data retrieving and analysis processes. Similar to TG-GATES studies, four to six male, 12 week old F344 rats were treated with low-, mid-, and high-dose of the toxicant and sacrificed at 6, 24 and 48 h later. At necropsy, liver was harvested for RNA extraction, histopathology, and clinical chemistry assessments^17^.

### Normalization and initial feature reduction by differential gene expression

To select best dose and earliest time point of liver toxicant exposure, EE data was used as described before^21–23^. Briefly, EE treatment data from the common array platform, Affymetrix Rat 230 2.0 microarray, which reported expression value of 31,099 genes were obtained from TG-GATES database. Data were normalized using the robust multi-array (RMA) average expression measure (Affy (v 1.57.0) package from Bioconductor)^24,25^. RMA was calculated on raw microarray gene expression values under standard normalization options (https://web.mit.edu/~r/current/arch/i386_linux26/lib/R/library/affy/html/AffyBatch-class.html). After normalization, the data were centered and scaled for differentially expressed genes analysis. To identify differentially expressed genes upon EE treatment, statistical analyses were performed on normalized gene expressions from dose response and time course data using Limma (v 3.34.9) package from Bioconductor^26,27^. Design matrices, constructed in R, identified coefficients of interest specifically high dose treatments (denoted with a 1) and control dose treatments (denoted with − 1). Gene expression data were first fitted to a multiple linear model, based on the design matrix. The linear model was then fitted to an empirical Bayes model with the contrast matrix representing the differences between high and control doses for each molecule^26,28,29^. T statistics and F-statistics were computed from the model. Significant features were selected with p-value < 0.05 for further feature selection methods. Resulting differentially expressed gene list was used to perform hierarchal clustering using Cluster 3 software^30^. Clustered data was visualized using Treeview java (https://jtreeview.sourceforge.net/). Gene set enrichment analysis software was used to identify enriched functional gene groupings^31,32^. Principal component analysis was performed using StrandNGS (Version 3.1.1, Bangalore, India). Graphs for biochemical analysis (blood alkaline phosphatase levels, total biluribin, body weight, liver weight and triglyceride levels) and average gene expression values were plotted using Graphpad Prism8 software (GraphPad Software Inc., La Jolla, CA, www.graphpad.com).

To prepare data for feature selection and classification using machine learning, microarray data (Affymetrix Rat 230 2.0) for compounds that induce necrosis were obtained from TG-GATES database and MAQ CII project. To avoid batch effects, data were normalized using the robust multi-array (RMA) average expression measure (Affy (v 1.57.0) package from Bioconductor)^24,25^. RMA was calculated on raw microarray gene expression values under standard normalization options (https://web.mit.edu/~r/current/arch/i386_linux26/lib/R/library/affy/html/AffyBatch-class.html). After normalization, the data were centered and scaled for gene expression analysis.

### Feature selection and comparative supervised machine learning

To assess the hypothesis that an early exposure gene signature is associated with liver toxicity, we applied a methodology^33,34^ that combines traditional statistical modeling with machine learning methods to perform predictor selection and ranking. These selected biomarkers formed the inputs for an integrative modeling process to determine the performance of significant markers for classification.

We integrated all analytical steps into a machine learning pipeline, similar to one used previously for patient classification^35–38^, as outlined below and summarized in Supplementary Fig. 1C.

First, to determine a gene feature’s measure of importance in predicting the necrosis response we used a set of feature selection approaches (marginal screening, embedded, and wrapper) on all predictors (i.e. genes and liver phenotypes)^39^ and an empirical ranking score based on the feature importance measure^34,40^. Methods for feature selection included Mann–Whitney, t-test, DCor as marginal screening methods; Boruta, RFE with both RF and SVM as wrapper methods; and RF, Elastic Net, Lasso, Ridge Regression Cross Validation (RidgeCV) and SVM as embedded methods. For each approach, the top N features were noted and utilized in the outer cross-validation loop of the integrative modeling process. Most algorithms are part of scikit-learn, scipy, and BorutaPy packages.

Cross-validation (out-of-sampling-testing) is utilized for obtaining the rankings by assessing every feature’s predictive power on unseen data^41,42^ with all compounds grouped together in the same fold and with a validation set^43^. Models were built for each feature selection approach and each predictive modeling approach. Predictive statistics were gathered as well as receiver operator characteristic (ROC) curves for each combination to visualize the classification performance (true positive rate vs. false positive rate) of the classifiers. Predictive modeling approaches include: logistic regression, RF, and support vector machine (SVM), Lasso and ElasticNet^36,44–46^. We built models incrementally from one feature to 100 features to understand and determine tradeoffs for identifying a cutoff for how many N features to select.

### Performance evaluation

Parameter tuning and performance evaluation were performed using the MAQCII-NIEHS (GSE16716) as the validation set, utilizing the area under the cross-validated ROC curve (AUC) as a quantitative performance metric. For parameter tuning, we tested tree depth of Boruta at 4, 5, 6, and no limit. We chose to focus on the depth of 4 to avoid overfitting. We experimented with alpha values for Elastic Net and Lasso using the Scikit learn GridSearchCV, which selects the best performing parameters. In addition, we experimented with the C value for SVC. For the rest of the algorithm default parameters were used. All parameters are listed in Table 2. Cross-validation^47^ partitions the samples into training and testing sets and proceed by fitting the model on the training set and evaluating the AUC on the testing set. Repeatedly performing the procedure independently, we summarize AUCs of all iterations for comparison^48^. To compare the performances of the developed classification model using gene biomarkers and the traditional diagnostic model, we obtained the AUC measures from all models over all randomization runs, and perform a two-sample t-test to detect differences. For each feature selection and classification method combinations, we reported area under the curve (AUC), F-statistics and MCC^49^ (Table 3). Results are visualized using Tableau software (Seattle, WA, USA, https://www.tableau.com/).

**Table 2:** Parameters used in each method.

Feature selection algorithms	
Algorithm	Parameters
ttest	Default parameters
Mann_Whitney	Default parameters
DCor	Default parameters
Boruta	{perc: 100, max_iter: 100, n_estimators: 15,000, max_depth: 6}
Lasso	{alpha: 0.001, max_iter: 20,000}
Lasso	{alpha: 0.01, max_iter: 20,000}
ElasticNet	{l1_ratio: 0.5, max_iter: 20,000, alpha: 0.001}
ElasticNet	{l1_ratio: 0.5, max_iter: 20,000, alpha: 0.01}
RandomForestClassifier	{n_estimators: 10,000, max_depth: null}
RidgeCV	default parameters
SVM(SVC)	{kernel: linear, C: 1}
Recursive feature selection with random forest	{n_estimators: 500, max_depth: null}
Recursive feature selection with SVM (SVC)	{kernel = linear}

Class prediction algorithms	
Algorithm	Parameters
RandomForestClassifier	{n_estimators = 1000, max_depth = 4}
SVC	{C = 1, kernel = 'linear'}
LogisticRegression	{max_iter = 20,000}
Lasso	{max_iter = 20,000, alpha = .001}
ElasticNet	{max_iter = 20,000, alpha = .001, l1_ratio = .5}

**Table 3:** Performance metric statistics of each feature selection-prediction method combination.

FS_name	Pred_method	nfold	mse	roc_auc	roc_auc_prob	Accuracy	f1_score	Precision_score	Recall_score	Sensitivity	Specificity	mcc
Boruta	RandomForest	Validation	0.102941	0.897059	0.933391	0.897059	0.895956	0.914634	0.897059	0.794118	1	0.811503
Boruta	RandomForest	0	0.090909	0.823529	0.895425	0.909091	0.903813	0.909091	0.909091	0.666667	0.980392	0.727607
Boruta	RandomForest	1	0.19697	0.707843	0.866667	0.80303	0.80059	0.798576	0.80303	0.533333	0.882353	0.426119
Boruta	RandomForest	2	0.092593	0.910714	0.950397	0.907407	0.910837	0.920798	0.907407	0.916667	0.904762	0.762443
Boruta	RandomForest	3	0.12963	0.857143	0.934524	0.87037	0.875171	0.88604	0.87037	0.833333	0.880952	0.662994
Boruta	RandomForest	4	0.148148	0.815476	0.863095	0.851852	0.855743	0.862302	0.851852	0.75	0.880952	0.598574
Boruta	RandomForest	5	0.185185	0.791667	0.865079	0.814815	0.823413	0.841374	0.814815	0.75	0.833333	0.531105
Boruta	RandomForest	6	0.407407	0.410714	0.605159	0.592593	0.592593	0.592593	0.592593	0.083333	0.738095	− 0.17857
Boruta	RandomForest	7	0.203704	0.660714	0.855159	0.796296	0.785258	0.780247	0.796296	0.416667	0.904762	0.358569
Boruta	RandomForest	8	0.111111	0.839286	0.865079	0.888889	0.888889	0.888889	0.888889	0.75	0.928571	0.678571
Boruta	RandomForest	9	0.092593	0.880952	0.954365	0.907407	0.908701	0.910778	0.907407	0.833333	0.928571	0.740888
Boruta	SVC	Validation	0.117647	0.882353	0.947232	0.882353	0.882251	0.883681	0.882353	0.852941	0.911765	0.766032
Boruta	SVC	0	0.166667	0.727451	0.878431	0.833333	0.826455	0.824074	0.833333	0.533333	0.921569	0.494266
Boruta	SVC	1	0.19697	0.731373	0.870588	0.80303	0.805232	0.807841	0.80303	0.6	0.862745	0.452509
Boruta	SVC	2	0.092593	0.910714	0.968254	0.907407	0.910837	0.920798	0.907407	0.916667	0.904762	0.762443
Boruta	SVC	3	0.185185	0.821429	0.902778	0.814815	0.826211	0.858025	0.814815	0.833333	0.809524	0.566947
Boruta	SVC	4	0.111111	0.89881	0.894841	0.888889	0.894048	0.910088	0.888889	0.916667	0.880952	0.726205
Boruta	SVC	5	0.203704	0.779762	0.871032	0.796296	0.807411	0.832362	0.796296	0.75	0.809524	0.500851
Boruta	SVC	6	0.388889	0.452381	0.460317	0.611111	0.616546	0.622264	0.611111	0.166667	0.738095	− 0.09261
Boruta	SVC	7	0.185185	0.732143	0.793651	0.814815	0.814815	0.814815	0.814815	0.583333	0.880952	0.464286
Boruta	SVC	8	0.185185	0.702381	0.809524	0.814815	0.808551	0.805051	0.814815	0.5	0.904762	0.4332
Boruta	SVC	9	0.148148	0.815476	0.890873	0.851852	0.855743	0.862302	0.851852	0.75	0.880952	0.598574
Boruta	LogisticRegression	Validation	0.161765	0.838235	0.944637	0.838235	0.835351	0.863721	0.838235	0.705882	0.970588	0.701493
Boruta	LogisticRegression	0	0.166667	0.633333	0.890196	0.833333	0.7932	0.862903	0.833333	0.266667	1	0.468353
Boruta	LogisticRegression	1	0.166667	0.703922	0.861438	0.833333	0.820561	0.821429	0.833333	0.466667	0.941176	0.476683
Boruta	LogisticRegression	2	0.111111	0.75	0.97619	0.888889	0.874074	0.902778	0.888889	0.5	1	0.661438
Boruta	LogisticRegression	3	0.074074	0.863095	0.888889	0.925926	0.92342	0.924747	0.925926	0.75	0.97619	0.777212
Boruta	LogisticRegression	4	0.166667	0.714286	0.894841	0.833333	0.824302	0.822222	0.833333	0.5	0.928571	0.478091
Boruta	LogisticRegression	5	0.148148	0.815476	0.833333	0.851852	0.855743	0.862302	0.851852	0.75	0.880952	0.598574
Boruta	LogisticRegression	6	0.277778	0.52381	0.470238	0.722222	0.693475	0.675785	0.722222	0.166667	0.880952	0.058938
Boruta	LogisticRegression	7	0.166667	0.684524	0.751984	0.833333	0.816085	0.820669	0.833333	0.416667	0.952381	0.456772
Boruta	LogisticRegression	8	0.277778	0.464286	0.728175	0.722222	0.65233	0.594771	0.722222	0	0.928571	− 0.12964
Boruta	LogisticRegression	9	0.166667	0.714286	0.875	0.833333	0.824302	0.822222	0.833333	0.5	0.928571	0.478091
Boruta	Lasso	Validation	0.176471	0.823529	0.943772	0.823529	0.819629	0.854167	0.823529	0.676471	0.970588	0.677003
Boruta	Lasso	0	0.166667	0.633333	0.870588	0.833333	0.7932	0.862903	0.833333	0.266667	1	0.468353
Boruta	Lasso	1	0.166667	0.703922	0.867974	0.833333	0.820561	0.821429	0.833333	0.466667	0.941176	0.476683
Boruta	Lasso	2	0.111111	0.75	0.96627	0.888889	0.874074	0.902778	0.888889	0.5	1	0.661438
Boruta	Lasso	3	0.111111	0.779762	0.896825	0.888889	0.880303	0.887681	0.888889	0.583333	0.97619	0.654802
Boruta	Lasso	4	0.185185	0.642857	0.896825	0.814815	0.790123	0.796296	0.814815	0.333333	0.952381	0.377964
Boruta	Lasso	5	0.148148	0.785714	0.84127	0.851852	0.851852	0.851852	0.851852	0.666667	0.904762	0.571429
Boruta	Lasso	6	0.240741	0.517857	0.494048	0.759259	0.698686	0.684096	0.759259	0.083333	0.952381	0.06482
Boruta	Lasso	7	0.185185	0.642857	0.761905	0.814815	0.790123	0.796296	0.814815	0.333333	0.952381	0.377964
Boruta	Lasso	8	0.277778	0.464286	0.769841	0.722222	0.65233	0.594771	0.722222	0	0.928571	− 0.12964
Boruta	Lasso	9	0.185185	0.672619	0.878968	0.814815	0.800505	0.798309	0.814815	0.416667	0.928571	0.404027
Boruta	ElasticNet	Validation	0.176471	0.823529	0.942042	0.823529	0.819629	0.854167	0.823529	0.676471	0.970588	0.677003
Boruta	ElasticNet	0	0.166667	0.633333	0.870588	0.833333	0.7932	0.862903	0.833333	0.266667	1	0.468353
Boruta	ElasticNet	1	0.166667	0.703922	0.869281	0.833333	0.820561	0.821429	0.833333	0.466667	0.941176	0.476683
Boruta	ElasticNet	2	0.111111	0.75	0.96627	0.888889	0.874074	0.902778	0.888889	0.5	1	0.661438
Boruta	ElasticNet	3	0.111111	0.779762	0.890873	0.888889	0.880303	0.887681	0.888889	0.583333	0.97619	0.654802
Boruta	ElasticNet	4	0.185185	0.642857	0.894841	0.814815	0.790123	0.796296	0.814815	0.333333	0.952381	0.377964
Boruta	ElasticNet	5	0.148148	0.785714	0.845238	0.851852	0.851852	0.851852	0.851852	0.666667	0.904762	0.571429
Boruta	ElasticNet	6	0.240741	0.517857	0.496032	0.759259	0.698686	0.684096	0.759259	0.083333	0.952381	0.06482
Boruta	ElasticNet	7	0.185185	0.642857	0.761905	0.814815	0.790123	0.796296	0.814815	0.333333	0.952381	0.377964
Boruta	ElasticNet	8	0.277778	0.464286	0.757937	0.722222	0.65233	0.594771	0.722222	0	0.928571	− 0.12964
Boruta	ElasticNet	9	0.185185	0.672619	0.880952	0.814815	0.800505	0.798309	0.814815	0.416667	0.928571	0.404027
DCor	RandomForest	Validation	0.102941	0.897059	0.916955	0.897059	0.896499	0.905836	0.897059	0.823529	0.970588	0.802846
DCor	RandomForest	0	0.106061	0.790196	0.917647	0.893939	0.885811	0.894481	0.893939	0.6	0.980392	0.678357
DCor	RandomForest	1	0.212121	0.65098	0.696732	0.787879	0.775564	0.770248	0.787879	0.4	0.901961	0.33955
DCor	RandomForest	2	0.148148	0.785714	0.938492	0.851852	0.851852	0.851852	0.851852	0.666667	0.904762	0.571429
DCor	RandomForest	3	0.203704	0.720238	0.875	0.796296	0.799143	0.802585	0.796296	0.583333	0.857143	0.428326
DCor	RandomForest	4	0.203704	0.720238	0.865079	0.796296	0.799143	0.802585	0.796296	0.583333	0.857143	0.428326
DCor	RandomForest	5	0.185185	0.791667	0.791667	0.814815	0.823413	0.841374	0.814815	0.75	0.833333	0.531105
DCor	RandomForest	6	0.425926	0.39881	0.380952	0.574074	0.580027	0.5862	0.574074	0.083333	0.714286	− 0.1968
DCor	RandomForest	7	0.203704	0.660714	0.857143	0.796296	0.785258	0.780247	0.796296	0.416667	0.904762	0.358569
DCor	RandomForest	8	0.092593	0.880952	0.886905	0.907407	0.908701	0.910778	0.907407	0.833333	0.928571	0.740888
DCor	RandomForest	9	0.111111	0.809524	0.958333	0.888889	0.88513	0.884848	0.888889	0.666667	0.952381	0.662541
DCor	SVC	Validation	0.132353	0.867647	0.933391	0.867647	0.867618	0.867965	0.867647	0.882353	0.852941	0.735612
DCor	SVC	0	0.121212	0.803922	0.881046	0.878788	0.875624	0.874654	0.878788	0.666667	0.941176	0.64049
DCor	SVC	1	0.212121	0.698039	0.79085	0.787879	0.787879	0.787879	0.787879	0.533333	0.862745	0.396078
DCor	SVC	2	0.166667	0.803571	0.914683	0.833333	0.839506	0.851282	0.833333	0.75	0.857143	0.563545
DCor	SVC	3	0.203704	0.779762	0.859127	0.796296	0.807411	0.832362	0.796296	0.75	0.809524	0.500851
DCor	SVC	4	0.203704	0.75	0.791667	0.796296	0.803841	0.816524	0.796296	0.666667	0.833333	0.464095
DCor	SVC	5	0.166667	0.803571	0.753968	0.833333	0.839506	0.851282	0.833333	0.75	0.857143	0.563545
DCor	SVC	6	0.481481	0.363095	0.337302	0.518519	0.540873	0.56652	0.518519	0.083333	0.642857	− 0.24929
DCor	SVC	7	0.203704	0.660714	0.777778	0.796296	0.785258	0.780247	0.796296	0.416667	0.904762	0.358569
DCor	SVC	8	0.12963	0.857143	0.934524	0.87037	0.875171	0.88604	0.87037	0.833333	0.880952	0.662994
DCor	SVC	9	0.111111	0.839286	0.962302	0.888889	0.888889	0.888889	0.888889	0.75	0.928571	0.678571
DCor	LogisticRegression	Validation	0.132353	0.867647	0.949827	0.867647	0.865287	0.895349	0.867647	0.735294	1	0.762493
DCor	LogisticRegression	0	0.151515	0.690196	0.909804	0.848485	0.826446	0.849659	0.848485	0.4	0.980392	0.517711
DCor	LogisticRegression	1	0.181818	0.670588	0.797386	0.818182	0.800505	0.802233	0.818182	0.4	0.941176	0.416631
DCor	LogisticRegression	2	0.148148	0.755952	0.914683	0.851852	0.84684	0.844949	0.851852	0.583333	0.928571	0.547871
DCor	LogisticRegression	3	0.185185	0.613095	0.85119	0.814815	0.77657	0.804444	0.814815	0.25	0.97619	0.359066
DCor	LogisticRegression	4	0.203704	0.660714	0.777778	0.796296	0.785258	0.780247	0.796296	0.416667	0.904762	0.358569
DCor	LogisticRegression	5	0.222222	0.64881	0.78373	0.777778	0.770261	0.765152	0.777778	0.416667	0.880952	0.318529
DCor	LogisticRegression	6	0.351852	0.446429	0.390873	0.648148	0.629082	0.612346	0.648148	0.083333	0.809524	− 0.11952
DCor	LogisticRegression	7	0.166667	0.625	0.789683	0.833333	0.791398	0.862745	0.833333	0.25	1	0.453743
DCor	LogisticRegression	8	0.166667	0.684524	0.926587	0.833333	0.816085	0.820669	0.833333	0.416667	0.952381	0.456772
DCor	LogisticRegression	9	0.12963	0.738095	0.94246	0.87037	0.856955	0.868963	0.87037	0.5	0.97619	0.589384
DCor	Lasso	Validation	0.132353	0.867647	0.948962	0.867647	0.865287	0.895349	0.867647	0.735294	1	0.762493
DCor	Lasso	0	0.181818	0.623529	0.895425	0.818182	0.780844	0.815201	0.818182	0.266667	0.980392	0.391274
DCor	Lasso	1	0.19697	0.637255	0.806536	0.80303	0.779381	0.781544	0.80303	0.333333	0.941176	0.352476
DCor	Lasso	2	0.166667	0.714286	0.936508	0.833333	0.824302	0.822222	0.833333	0.5	0.928571	0.478091
DCor	Lasso	3	0.222222	0.529762	0.867063	0.777778	0.710233	0.724359	0.777778	0.083333	0.97619	0.131036
DCor	Lasso	4	0.222222	0.619048	0.777778	0.777778	0.760606	0.753623	0.777778	0.333333	0.904762	0.278639
DCor	Lasso	5	0.222222	0.64881	0.787698	0.777778	0.770261	0.765152	0.777778	0.416667	0.880952	0.318529
DCor	Lasso	6	0.259259	0.505952	0.369048	0.740741	0.687198	0.662222	0.740741	0.083333	0.928571	0.018898
DCor	Lasso	7	0.166667	0.625	0.797619	0.833333	0.791398	0.862745	0.833333	0.25	1	0.453743
DCor	Lasso	8	0.12963	0.738095	0.928571	0.87037	0.856955	0.868963	0.87037	0.5	0.97619	0.589384
DCor	Lasso	9	0.111111	0.75	0.930556	0.888889	0.874074	0.902778	0.888889	0.5	1	0.661438
DCor	ElasticNet	Validation	0.132353	0.867647	0.948097	0.867647	0.865287	0.895349	0.867647	0.735294	1	0.762493
DCor	ElasticNet	0	0.181818	0.623529	0.89281	0.818182	0.780844	0.815201	0.818182	0.266667	0.980392	0.391274
DCor	ElasticNet	1	0.19697	0.637255	0.803922	0.80303	0.779381	0.781544	0.80303	0.333333	0.941176	0.352476
DCor	ElasticNet	2	0.166667	0.714286	0.936508	0.833333	0.824302	0.822222	0.833333	0.5	0.928571	0.478091
DCor	ElasticNet	3	0.222222	0.529762	0.867063	0.777778	0.710233	0.724359	0.777778	0.083333	0.97619	0.131036
DCor	ElasticNet	4	0.203704	0.660714	0.779762	0.796296	0.785258	0.780247	0.796296	0.416667	0.904762	0.358569
DCor	ElasticNet	5	0.222222	0.64881	0.78373	0.777778	0.770261	0.765152	0.777778	0.416667	0.880952	0.318529
DCor	ElasticNet	6	0.277778	0.494048	0.365079	0.722222	0.675716	0.647619	0.722222	0.083333	0.904762	− 0.01708
DCor	ElasticNet	7	0.166667	0.625	0.793651	0.833333	0.791398	0.862745	0.833333	0.25	1	0.453743
DCor	ElasticNet	8	0.148148	0.696429	0.926587	0.851852	0.832099	0.849537	0.851852	0.416667	0.97619	0.519701
DCor	ElasticNet	9	0.111111	0.75	0.938492	0.888889	0.874074	0.902778	0.888889	0.5	1	0.661438
ElasticNet_alpha_.001	RandomForest	Validation	0.279412	0.720588	0.75519	0.720588	0.719069	0.725464	0.720588	0.794118	0.647059	0.446026
ElasticNet_alpha_.001	RandomForest	0	0.227273	0.594118	0.620915	0.772727	0.74544	0.739812	0.772727	0.266667	0.921569	0.241698
ElasticNet_alpha_.001	RandomForest	1	0.318182	0.558824	0.670588	0.681818	0.685375	0.689205	0.681818	0.333333	0.784314	0.115045
ElasticNet_alpha_.001	RandomForest	2	0.240741	0.636905	0.734127	0.759259	0.755442	0.752173	0.759259	0.416667	0.857143	0.28264
ElasticNet_alpha_.001	RandomForest	3	0.240741	0.666667	0.765873	0.759259	0.762624	0.766521	0.759259	0.5	0.833333	0.324138
ElasticNet_alpha_.001	RandomForest	4	0.222222	0.738095	0.77381	0.777778	0.788095	0.807018	0.777778	0.666667	0.809524	0.433555
ElasticNet_alpha_.001	RandomForest	5	0.166667	0.77381	0.934524	0.833333	0.835663	0.838649	0.833333	0.666667	0.880952	0.532513
ElasticNet_alpha_.001	RandomForest	6	0.240741	0.577381	0.53373	0.759259	0.734345	0.72408	0.759259	0.25	0.904762	0.19155
ElasticNet_alpha_.001	RandomForest	7	0.111111	0.779762	0.875	0.888889	0.880303	0.887681	0.888889	0.583333	0.97619	0.654802
ElasticNet_alpha_.001	RandomForest	8	0.203704	0.630952	0.700397	0.796296	0.775215	0.772374	0.796296	0.333333	0.928571	0.324161
ElasticNet_alpha_.001	RandomForest	9	0.166667	0.744048	0.779762	0.833333	0.830691	0.828753	0.833333	0.583333	0.904762	0.503836
ElasticNet_alpha_.001	SVC	Validation	0.25	0.75	0.762111	0.75	0.748641	0.755526	0.75	0.676471	0.823529	0.505496
ElasticNet_alpha_.001	SVC	0	0.287879	0.672549	0.687582	0.712121	0.728747	0.760331	0.712121	0.6	0.745098	0.306786
ElasticNet_alpha_.001	SVC	1	0.287879	0.64902	0.708497	0.712121	0.725264	0.746047	0.712121	0.533333	0.764706	0.271775
ElasticNet_alpha_.001	SVC	2	0.37037	0.613095	0.603175	0.62963	0.659071	0.726957	0.62963	0.583333	0.642857	0.191383
ElasticNet_alpha_.001	SVC	3	0.259259	0.654762	0.746032	0.740741	0.747551	0.756349	0.740741	0.5	0.809524	0.29364
ElasticNet_alpha_.001	SVC	4	0.314815	0.708333	0.775794	0.685185	0.710937	0.789465	0.685185	0.75	0.666667	0.350315
ElasticNet_alpha_.001	SVC	5	0.203704	0.839286	0.928571	0.796296	0.811852	0.870611	0.796296	0.916667	0.761905	0.578688
ElasticNet_alpha_.001	SVC	6	0.388889	0.541667	0.678571	0.611111	0.637341	0.680702	0.611111	0.416667	0.666667	0.072548
ElasticNet_alpha_.001	SVC	7	0.111111	0.89881	0.94246	0.888889	0.894048	0.910088	0.888889	0.916667	0.880952	0.726205
ElasticNet_alpha_.001	SVC	8	0.388889	0.571429	0.607143	0.611111	0.640808	0.699856	0.611111	0.5	0.642857	0.121829
ElasticNet_alpha_.001	SVC	9	0.333333	0.636905	0.777778	0.666667	0.690789	0.741176	0.666667	0.583333	0.690476	0.235727
ElasticNet_alpha_.001	LogisticRegression	Validation	0.220588	0.779412	0.741349	0.779412	0.771044	0.827254	0.779412	0.588235	0.970588	0.604777
ElasticNet_alpha_.001	LogisticRegression	0	0.19697	0.590196	0.695425	0.80303	0.7556	0.793622	0.80303	0.2	0.980392	0.316827
ElasticNet_alpha_.001	LogisticRegression	1	0.257576	0.55098	0.70719	0.742424	0.711499	0.69808	0.742424	0.2	0.901961	0.13092
ElasticNet_alpha_.001	LogisticRegression	2	0.259259	0.535714	0.619048	0.740741	0.706173	0.689815	0.740741	0.166667	0.904762	0.094491
ElasticNet_alpha_.001	LogisticRegression	3	0.203704	0.541667	0.797619	0.796296	0.721907	0.838574	0.796296	0.083333	1	0.256978
ElasticNet_alpha_.001	LogisticRegression	4	0.203704	0.690476	0.771825	0.796296	0.793066	0.790463	0.796296	0.5	0.880952	0.393238
ElasticNet_alpha_.001	LogisticRegression	5	0.074074	0.833333	0.944444	0.925926	0.920202	0.932367	0.925926	0.666667	1	0.780189
ElasticNet_alpha_.001	LogisticRegression	6	0.185185	0.583333	0.674603	0.814815	0.758528	0.850427	0.814815	0.166667	1	0.3669
ElasticNet_alpha_.001	LogisticRegression	7	0.166667	0.654762	0.904762	0.833333	0.80543	0.828571	0.833333	0.333333	0.97619	0.443942
ElasticNet_alpha_.001	LogisticRegression	8	0.185185	0.583333	0.609127	0.814815	0.758528	0.850427	0.814815	0.166667	1	0.3669
ElasticNet_alpha_.001	LogisticRegression	9	0.148148	0.666667	0.803571	0.851852	0.821256	0.875556	0.851852	0.333333	1	0.52915
ElasticNet_alpha_.001	Lasso	Validation	0.235294	0.764706	0.736159	0.764706	0.759505	0.789773	0.764706	0.617647	0.911765	0.553912
ElasticNet_alpha_.001	Lasso	0	0.227273	0.523529	0.718954	0.772727	0.698675	0.71733	0.772727	0.066667	0.980392	0.115045
ElasticNet_alpha_.001	Lasso	1	0.212121	0.580392	0.705882	0.787879	0.744318	0.757079	0.787879	0.2	0.960784	0.254639
ElasticNet_alpha_.001	Lasso	2	0.240741	0.547619	0.603175	0.759259	0.718954	0.707937	0.759259	0.166667	0.928571	0.136598
ElasticNet_alpha_.001	Lasso	3	0.203704	0.541667	0.815476	0.796296	0.721907	0.838574	0.796296	0.083333	1	0.256978
ElasticNet_alpha_.001	Lasso	4	0.166667	0.714286	0.777778	0.833333	0.824302	0.822222	0.833333	0.5	0.928571	0.478091
ElasticNet_alpha_.001	Lasso	5	0.092593	0.791667	0.938492	0.907407	0.897825	0.917258	0.907407	0.583333	1	0.721995
ElasticNet_alpha_.001	Lasso	6	0.185185	0.583333	0.68254	0.814815	0.758528	0.850427	0.814815	0.166667	1	0.3669
ElasticNet_alpha_.001	Lasso	7	0.166667	0.654762	0.914683	0.833333	0.80543	0.828571	0.833333	0.333333	0.97619	0.443942
ElasticNet_alpha_.001	Lasso	8	0.185185	0.583333	0.625	0.814815	0.758528	0.850427	0.814815	0.166667	1	0.3669
ElasticNet_alpha_.001	Lasso	9	0.148148	0.666667	0.785714	0.851852	0.821256	0.875556	0.851852	0.333333	1	0.52915
ElasticNet_alpha_.001	ElasticNet	Validation	0.235294	0.764706	0.734429	0.764706	0.759505	0.789773	0.764706	0.617647	0.911765	0.553912
ElasticNet_alpha_.001	ElasticNet	0	0.212121	0.556863	0.720261	0.787879	0.728336	0.764791	0.787879	0.133333	0.980392	0.228801
ElasticNet_alpha_.001	ElasticNet	1	0.212121	0.580392	0.70719	0.787879	0.744318	0.757079	0.787879	0.2	0.960784	0.254639
ElasticNet_alpha_.001	ElasticNet	2	0.240741	0.547619	0.599206	0.759259	0.718954	0.707937	0.759259	0.166667	0.928571	0.136598
ElasticNet_alpha_.001	ElasticNet	3	0.203704	0.541667	0.811508	0.796296	0.721907	0.838574	0.796296	0.083333	1	0.256978
ElasticNet_alpha_.001	ElasticNet	4	0.166667	0.714286	0.77381	0.833333	0.824302	0.822222	0.833333	0.5	0.928571	0.478091
ElasticNet_alpha_.001	ElasticNet	5	0.092593	0.791667	0.938492	0.907407	0.897825	0.917258	0.907407	0.583333	1	0.721995
ElasticNet_alpha_.001	ElasticNet	6	0.185185	0.583333	0.688492	0.814815	0.758528	0.850427	0.814815	0.166667	1	0.3669
ElasticNet_alpha_.001	ElasticNet	7	0.166667	0.654762	0.910714	0.833333	0.80543	0.828571	0.833333	0.333333	0.97619	0.443942
ElasticNet_alpha_.001	ElasticNet	8	0.185185	0.583333	0.611111	0.814815	0.758528	0.850427	0.814815	0.166667	1	0.3669
ElasticNet_alpha_.001	ElasticNet	9	0.148148	0.666667	0.785714	0.851852	0.821256	0.875556	0.851852	0.333333	1	0.52915
ElasticNet_alpha_.01	RandomForest	Validation	0.397059	0.602941	0.676471	0.602941	0.587879	0.620567	0.602941	0.794118	0.411765	0.222812
ElasticNet_alpha_.01	RandomForest	0	0.242424	0.490196	0.605229	0.757576	0.666144	0.594406	0.757576	0	0.980392	− 0.06727
ElasticNet_alpha_.01	RandomForest	1	0.227273	0.688235	0.720261	0.772727	0.775268	0.778182	0.772727	0.533333	0.843137	0.368143
ElasticNet_alpha_.01	RandomForest	2	0.092593	0.791667	0.972222	0.907407	0.897825	0.917258	0.907407	0.583333	1	0.721995
ElasticNet_alpha_.01	RandomForest	3	0.074074	0.833333	0.880952	0.925926	0.920202	0.932367	0.925926	0.666667	1	0.780189
ElasticNet_alpha_.01	RandomForest	4	0.092593	0.821429	0.875	0.907407	0.90239	0.906173	0.907407	0.666667	0.97619	0.717137
ElasticNet_alpha_.01	RandomForest	5	0.055556	0.904762	0.980159	0.944444	0.943564	0.943622	0.944444	0.833333	0.97619	0.835631
ElasticNet_alpha_.01	RandomForest	6	0.203704	0.60119	0.65873	0.796296	0.762192	0.768254	0.796296	0.25	0.952381	0.29027
ElasticNet_alpha_.01	RandomForest	7	0.222222	0.678571	0.829365	0.777778	0.777778	0.777778	0.777778	0.5	0.857143	0.357143
ElasticNet_alpha_.01	RandomForest	8	0.277778	0.494048	0.684524	0.722222	0.675716	0.647619	0.722222	0.083333	0.904762	− 0.01708
ElasticNet_alpha_.01	RandomForest	9	0.074074	0.863095	0.871032	0.925926	0.92342	0.924747	0.925926	0.75	0.97619	0.777212
ElasticNet_alpha_.01	SVC	Validation	0.5	0.5	0.545848	0.5	0.333333	0.25	0.5	1	0	0
ElasticNet_alpha_.01	SVC	0	0.257576	0.55098	0.751634	0.742424	0.711499	0.69808	0.742424	0.2	0.901961	0.13092
ElasticNet_alpha_.01	SVC	1	0.348485	0.609804	0.747712	0.651515	0.674863	0.719697	0.651515	0.533333	0.686275	0.191315
ElasticNet_alpha_.01	SVC	2	0.166667	0.803571	0.902778	0.833333	0.839506	0.851282	0.833333	0.75	0.857143	0.563545
ElasticNet_alpha_.01	SVC	3	0.259259	0.714286	0.795635	0.740741	0.756695	0.790123	0.740741	0.666667	0.761905	0.377964
ElasticNet_alpha_.01	SVC	4	0.222222	0.708333	0.849206	0.777778	0.783615	0.791667	0.777778	0.583333	0.833333	0.395285
ElasticNet_alpha_.01	SVC	5	0.148148	0.845238	0.950397	0.851852	0.85873	0.875731	0.851852	0.833333	0.857143	0.628655
ElasticNet_alpha_.01	SVC	6	0.203704	0.660714	0.805556	0.796296	0.785258	0.780247	0.796296	0.416667	0.904762	0.358569
ElasticNet_alpha_.01	SVC	7	0.203704	0.75	0.894841	0.796296	0.803841	0.816524	0.796296	0.666667	0.833333	0.464095
ElasticNet_alpha_.01	SVC	8	0.351852	0.446429	0.555556	0.648148	0.629082	0.612346	0.648148	0.083333	0.809524	−0.11952
ElasticNet_alpha_.01	SVC	9	0.259259	0.77381	0.865079	0.740741	0.76135	0.830177	0.740741	0.833333	0.714286	0.463348
ElasticNet_alpha_.01	LogisticRegression	Validation	0.588235	0.411765	0.553633	0.411765	0.328063	0.324138	0.411765	0.764706	0.058824	− 0.24914
ElasticNet_alpha_.01	LogisticRegression	0	0.227273	0.5	0.718954	0.772727	0.67366	0.597107	0.772727	0	1	0
ElasticNet_alpha_.01	LogisticRegression	1	0.227273	0.617647	0.766013	0.772727	0.75531	0.748377	0.772727	0.333333	0.901961	0.27501
ElasticNet_alpha_.01	LogisticRegression	2	0.111111	0.75	0.878968	0.888889	0.874074	0.902778	0.888889	0.5	1	0.661438
ElasticNet_alpha_.01	LogisticRegression	3	0.12963	0.738095	0.779762	0.87037	0.856955	0.868963	0.87037	0.5	0.97619	0.589384
ElasticNet_alpha_.01	LogisticRegression	4	0.148148	0.72619	0.837302	0.851852	0.840404	0.842995	0.851852	0.5	0.952381	0.529414
ElasticNet_alpha_.01	LogisticRegression	5	0.092593	0.821429	0.968254	0.907407	0.90239	0.906173	0.907407	0.666667	0.97619	0.717137
ElasticNet_alpha_.01	LogisticRegression	6	0.185185	0.583333	0.827381	0.814815	0.758528	0.850427	0.814815	0.166667	1	0.3669
ElasticNet_alpha_.01	LogisticRegression	7	0.12963	0.708333	0.888889	0.87037	0.848668	0.888889	0.87037	0.416667	1	0.597614
ElasticNet_alpha_.01	LogisticRegression	8	0.259259	0.47619	0.561508	0.740741	0.661939	0.598291	0.740741	0	0.952381	− 0.10483
ElasticNet_alpha_.01	LogisticRegression	9	0.074074	0.833333	0.871032	0.925926	0.920202	0.932367	0.925926	0.666667	1	0.780189
ElasticNet_alpha_.01	Lasso	Validation	0.558824	0.441176	0.562284	0.441176	0.416441	0.429167	0.441176	0.647059	0.235294	− 0.1291
ElasticNet_alpha_.01	Lasso	0	0.227273	0.5	0.729412	0.772727	0.67366	0.597107	0.772727	0	1	0
ElasticNet_alpha_.01	Lasso	1	0.212121	0.603922	0.762092	0.787879	0.757025	0.75853	0.787879	0.266667	0.941176	0.282873
ElasticNet_alpha_.01	Lasso	2	0.12963	0.708333	0.853175	0.87037	0.848668	0.888889	0.87037	0.416667	1	0.597614
ElasticNet_alpha_.01	Lasso	3	0.111111	0.75	0.771825	0.888889	0.874074	0.902778	0.888889	0.5	1	0.661438
ElasticNet_alpha_.01	Lasso	4	0.148148	0.696429	0.80754	0.851852	0.832099	0.849537	0.851852	0.416667	0.97619	0.519701
ElasticNet_alpha_.01	Lasso	5	0.074074	0.833333	0.960317	0.925926	0.920202	0.932367	0.925926	0.666667	1	0.780189
ElasticNet_alpha_.01	Lasso	6	0.185185	0.583333	0.803571	0.814815	0.758528	0.850427	0.814815	0.166667	1	0.3669
ElasticNet_alpha_.01	Lasso	7	0.148148	0.666667	0.890873	0.851852	0.821256	0.875556	0.851852	0.333333	1	0.52915
ElasticNet_alpha_.01	Lasso	8	0.240741	0.488095	0.547619	0.759259	0.671345	0.601677	0.759259	0	0.97619	− 0.07342
ElasticNet_alpha_.01	Lasso	9	0.092593	0.791667	0.876984	0.907407	0.897825	0.917258	0.907407	0.583333	1	0.721995
ElasticNet_alpha_.01	ElasticNet	Validation	0.558824	0.441176	0.557958	0.441176	0.416441	0.429167	0.441176	0.647059	0.235294	− 0.1291
ElasticNet_alpha_.01	ElasticNet	0	0.227273	0.5	0.730719	0.772727	0.67366	0.597107	0.772727	0	1	0
ElasticNet_alpha_.01	ElasticNet	1	0.212121	0.603922	0.760784	0.787879	0.757025	0.75853	0.787879	0.266667	0.941176	0.282873
ElasticNet_alpha_.01	ElasticNet	2	0.12963	0.708333	0.853175	0.87037	0.848668	0.888889	0.87037	0.416667	1	0.597614
ElasticNet_alpha_.01	ElasticNet	3	0.111111	0.75	0.77381	0.888889	0.874074	0.902778	0.888889	0.5	1	0.661438
ElasticNet_alpha_.01	ElasticNet	4	0.148148	0.696429	0.80754	0.851852	0.832099	0.849537	0.851852	0.416667	0.97619	0.519701
ElasticNet_alpha_.01	ElasticNet	5	0.074074	0.833333	0.958333	0.925926	0.920202	0.932367	0.925926	0.666667	1	0.780189
ElasticNet_alpha_.01	ElasticNet	6	0.185185	0.583333	0.801587	0.814815	0.758528	0.850427	0.814815	0.166667	1	0.3669
ElasticNet_alpha_.01	ElasticNet	7	0.148148	0.666667	0.888889	0.851852	0.821256	0.875556	0.851852	0.333333	1	0.52915
ElasticNet_alpha_.01	ElasticNet	8	0.240741	0.488095	0.559524	0.759259	0.671345	0.601677	0.759259	0	0.97619	− 0.07342
ElasticNet_alpha_.01	ElasticNet	9	0.092593	0.791667	0.876984	0.907407	0.897825	0.917258	0.907407	0.583333	1	0.721995
Lasso_alpha_.001	RandomForest	Validation	0.470588	0.529412	0.663495	0.529412	0.484848	0.544974	0.529412	0.823529	0.235294	0.072739
Lasso_alpha_.001	RandomForest	0	0.227273	0.570588	0.636601	0.772727	0.73324	0.731818	0.772727	0.2	0.941176	0.205798
Lasso_alpha_.001	RandomForest	1	0.227273	0.664706	0.733333	0.772727	0.769912	0.767483	0.772727	0.466667	0.862745	0.337679
Lasso_alpha_.001	RandomForest	2	0.185185	0.672619	0.75	0.814815	0.800505	0.798309	0.814815	0.416667	0.928571	0.404027
Lasso_alpha_.001	RandomForest	3	0.240741	0.607143	0.730159	0.759259	0.746214	0.738272	0.759259	0.333333	0.880952	0.239046
Lasso_alpha_.001	RandomForest	4	0.185185	0.761905	0.767857	0.814815	0.819679	0.826984	0.814815	0.666667	0.857143	0.496929
Lasso_alpha_.001	RandomForest	5	0.12963	0.797619	0.875	0.87037	0.868315	0.867043	0.87037	0.666667	0.928571	0.614434
Lasso_alpha_.001	RandomForest	6	0.222222	0.559524	0.664683	0.777778	0.731884	0.733333	0.777778	0.166667	0.952381	0.188982
Lasso_alpha_.001	RandomForest	7	0.092593	0.791667	0.775794	0.907407	0.897825	0.917258	0.907407	0.583333	1	0.721995
Lasso_alpha_.001	RandomForest	8	0.185185	0.613095	0.730159	0.814815	0.77657	0.804444	0.814815	0.25	0.97619	0.359066
Lasso_alpha_.001	RandomForest	9	0.277778	0.613095	0.732143	0.722222	0.726104	0.730457	0.722222	0.416667	0.809524	0.219951
Lasso_alpha_.001	SVC	Validation	0.573529	0.426471	0.605536	0.426471	0.366005	0.381119	0.426471	0.735294	0.117647	− 0.18699
Lasso_alpha_.001	SVC	0	0.257576	0.668627	0.695425	0.742424	0.75023	0.761048	0.742424	0.533333	0.803922	0.317345
Lasso_alpha_.001	SVC	1	0.287879	0.672549	0.739869	0.712121	0.728747	0.760331	0.712121	0.6	0.745098	0.306786
Lasso_alpha_.001	SVC	2	0.351852	0.595238	0.626984	0.648148	0.67188	0.71462	0.648148	0.5	0.690476	0.165823
Lasso_alpha_.001	SVC	3	0.388889	0.511905	0.613095	0.611111	0.632329	0.661897	0.611111	0.333333	0.690476	0.021313
Lasso_alpha_.001	SVC	4	0.296296	0.690476	0.744048	0.703704	0.725146	0.775163	0.703704	0.666667	0.714286	0.327968
Lasso_alpha_.001	SVC	5	0.166667	0.803571	0.884921	0.833333	0.839506	0.851282	0.833333	0.75	0.857143	0.563545
Lasso_alpha_.001	SVC	6	0.259259	0.744048	0.857143	0.740741	0.759503	0.80915	0.740741	0.75	0.738095	0.420209
Lasso_alpha_.001	SVC	7	0.222222	0.767857	0.880952	0.777778	0.791453	0.824074	0.777778	0.75	0.785714	0.472456
Lasso_alpha_.001	SVC	8	0.388889	0.541667	0.609127	0.611111	0.637341	0.680702	0.611111	0.416667	0.666667	0.072548
Lasso_alpha_.001	SVC	9	0.37037	0.583333	0.722222	0.62963	0.656433	0.70719	0.62963	0.5	0.666667	0.143486
Lasso_alpha_.001	LogisticRegression	Validation	0.367647	0.632353	0.627163	0.632353	0.631636	0.633391	0.632353	0.588235	0.676471	0.265742
Lasso_alpha_.001	LogisticRegression	0	0.19697	0.566667	0.695425	0.80303	0.738851	0.84304	0.80303	0.133333	1	0.32596
Lasso_alpha_.001	LogisticRegression	1	0.181818	0.623529	0.737255	0.818182	0.780844	0.815201	0.818182	0.266667	0.980392	0.391274
Lasso_alpha_.001	LogisticRegression	2	0.240741	0.547619	0.652778	0.759259	0.718954	0.707937	0.759259	0.166667	0.928571	0.136598
Lasso_alpha_.001	LogisticRegression	3	0.203704	0.541667	0.656746	0.796296	0.721907	0.838574	0.796296	0.083333	1	0.256978
Lasso_alpha_.001	LogisticRegression	4	0.166667	0.714286	0.738095	0.833333	0.824302	0.822222	0.833333	0.5	0.928571	0.478091
Lasso_alpha_.001	LogisticRegression	5	0.074074	0.833333	0.902778	0.925926	0.920202	0.932367	0.925926	0.666667	1	0.780189
Lasso_alpha_.001	LogisticRegression	6	0.185185	0.583333	0.855159	0.814815	0.758528	0.850427	0.814815	0.166667	1	0.3669
Lasso_alpha_.001	LogisticRegression	7	0.111111	0.75	0.84127	0.888889	0.874074	0.902778	0.888889	0.5	1	0.661438
Lasso_alpha_.001	LogisticRegression	8	0.185185	0.583333	0.626984	0.814815	0.758528	0.850427	0.814815	0.166667	1	0.3669
Lasso_alpha_.001	LogisticRegression	9	0.185185	0.672619	0.712302	0.814815	0.800505	0.798309	0.814815	0.416667	0.928571	0.404027
Lasso_alpha_.001	Lasso	Validation	0.426471	0.573529	0.605536	0.573529	0.573437	0.573593	0.573529	0.588235	0.558824	0.147122
Lasso_alpha_.001	Lasso	0	0.227273	0.5	0.729412	0.772727	0.67366	0.597107	0.772727	0	1	0
Lasso_alpha_.001	Lasso	1	0.181818	0.623529	0.717647	0.818182	0.780844	0.815201	0.818182	0.266667	0.980392	0.391274
Lasso_alpha_.001	Lasso	2	0.222222	0.559524	0.64881	0.777778	0.731884	0.733333	0.777778	0.166667	0.952381	0.188982
Lasso_alpha_.001	Lasso	3	0.203704	0.541667	0.706349	0.796296	0.721907	0.838574	0.796296	0.083333	1	0.256978
Lasso_alpha_.001	Lasso	4	0.185185	0.672619	0.730159	0.814815	0.800505	0.798309	0.814815	0.416667	0.928571	0.404027
Lasso_alpha_.001	Lasso	5	0.111111	0.75	0.888889	0.888889	0.874074	0.902778	0.888889	0.5	1	0.661438
Lasso_alpha_.001	Lasso	6	0.185185	0.583333	0.871032	0.814815	0.758528	0.850427	0.814815	0.166667	1	0.3669
Lasso_alpha_.001	Lasso	7	0.148148	0.666667	0.839286	0.851852	0.821256	0.875556	0.851852	0.333333	1	0.52915
Lasso_alpha_.001	Lasso	8	0.203704	0.541667	0.632937	0.796296	0.721907	0.838574	0.796296	0.083333	1	0.256978
Lasso_alpha_.001	Lasso	9	0.148148	0.666667	0.714286	0.851852	0.821256	0.875556	0.851852	0.333333	1	0.52915
Lasso_alpha_.001	ElasticNet	Validation	0.426471	0.573529	0.602941	0.573529	0.573437	0.573593	0.573529	0.588235	0.558824	0.147122
Lasso_alpha_.001	ElasticNet	0	0.212121	0.533333	0.734641	0.787879	0.707876	0.833566	0.787879	0.066667	1	0.228709
Lasso_alpha_.001	ElasticNet	1	0.181818	0.623529	0.720261	0.818182	0.780844	0.815201	0.818182	0.266667	0.980392	0.391274
Lasso_alpha_.001	ElasticNet	2	0.222222	0.559524	0.644841	0.777778	0.731884	0.733333	0.777778	0.166667	0.952381	0.188982
Lasso_alpha_.001	ElasticNet	3	0.203704	0.541667	0.702381	0.796296	0.721907	0.838574	0.796296	0.083333	1	0.256978
Lasso_alpha_.001	ElasticNet	4	0.185185	0.672619	0.730159	0.814815	0.800505	0.798309	0.814815	0.416667	0.928571	0.404027
Lasso_alpha_.001	ElasticNet	5	0.092593	0.791667	0.888889	0.907407	0.897825	0.917258	0.907407	0.583333	1	0.721995
Lasso_alpha_.001	ElasticNet	6	0.185185	0.583333	0.876984	0.814815	0.758528	0.850427	0.814815	0.166667	1	0.3669
Lasso_alpha_.001	ElasticNet	7	0.148148	0.666667	0.847222	0.851852	0.821256	0.875556	0.851852	0.333333	1	0.52915
Lasso_alpha_.001	ElasticNet	8	0.203704	0.541667	0.632937	0.796296	0.721907	0.838574	0.796296	0.083333	1	0.256978
Lasso_alpha_.001	ElasticNet	9	0.148148	0.666667	0.712302	0.851852	0.821256	0.875556	0.851852	0.333333	1	0.52915
Lasso_alpha_.01	RandomForest	Validation	0.514706	0.485294	0.474048	0.485294	0.326733	0.246269	0.485294	0.970588	0	− 0.12217
Lasso_alpha_.01	RandomForest	0	0.242424	0.537255	0.762092	0.757576	0.707792	0.698957	0.757576	0.133333	0.941176	0.118003
Lasso_alpha_.01	RandomForest	1	0.242424	0.678431	0.764706	0.757576	0.762727	0.76929	0.757576	0.533333	0.823529	0.341987
Lasso_alpha_.01	RandomForest	2	0.12963	0.738095	0.96627	0.87037	0.856955	0.868963	0.87037	0.5	0.97619	0.589384
Lasso_alpha_.01	RandomForest	3	0.12963	0.797619	0.956349	0.87037	0.868315	0.867043	0.87037	0.666667	0.928571	0.614434
Lasso_alpha_.01	RandomForest	4	0.092593	0.85119	0.934524	0.907407	0.905939	0.905332	0.907407	0.75	0.952381	0.725032
Lasso_alpha_.01	RandomForest	5	0.092593	0.910714	0.978175	0.907407	0.910837	0.920798	0.907407	0.916667	0.904762	0.762443
Lasso_alpha_.01	RandomForest	6	0.277778	0.553571	0.634921	0.722222	0.70717	0.696296	0.722222	0.25	0.857143	0.119523
Lasso_alpha_.01	RandomForest	7	0.222222	0.708333	0.730159	0.777778	0.783615	0.791667	0.777778	0.583333	0.833333	0.395285
Lasso_alpha_.01	RandomForest	8	0.277778	0.464286	0.767857	0.722222	0.65233	0.594771	0.722222	0	0.928571	− 0.12964
Lasso_alpha_.01	RandomForest	9	0.092593	0.821429	0.884921	0.907407	0.90239	0.906173	0.907407	0.666667	0.97619	0.717137
Lasso_alpha_.01	SVC	Validation	0.838235	0.161765	0.110727	0.161765	0.139241	0.122222	0.161765	0.323529	0	− 0.71492
Lasso_alpha_.01	SVC	0	0.19697	0.707843	0.779085	0.80303	0.80059	0.798576	0.80303	0.533333	0.882353	0.426119
Lasso_alpha_.01	SVC	1	0.227273	0.758824	0.844444	0.772727	0.785853	0.816116	0.772727	0.733333	0.784314	0.460179
Lasso_alpha_.01	SVC	2	0.240741	0.785714	0.900794	0.759259	0.777643	0.83646	0.759259	0.833333	0.738095	0.487316
Lasso_alpha_.01	SVC	3	0.185185	0.821429	0.912698	0.814815	0.826211	0.858025	0.814815	0.833333	0.809524	0.566947
Lasso_alpha_.01	SVC	4	0.148148	0.845238	0.928571	0.851852	0.85873	0.875731	0.851852	0.833333	0.857143	0.628655
Lasso_alpha_.01	SVC	5	0.037037	0.97619	0.996032	0.962963	0.963936	0.968254	0.962963	1	0.952381	0.903508
Lasso_alpha_.01	SVC	6	0.259259	0.625	0.779762	0.740741	0.740741	0.740741	0.740741	0.416667	0.833333	0.25
Lasso_alpha_.01	SVC	7	0.185185	0.761905	0.819444	0.814815	0.819679	0.826984	0.814815	0.666667	0.857143	0.496929
Lasso_alpha_.01	SVC	8	0.203704	0.690476	0.72619	0.796296	0.793066	0.790463	0.796296	0.5	0.880952	0.393238
Lasso_alpha_.01	SVC	9	0.185185	0.791667	0.900794	0.814815	0.823413	0.841374	0.814815	0.75	0.833333	0.531105
Lasso_alpha_.01	LogisticRegression	Validation	0.867647	0.132353	0.122837	0.132353	0.116883	0.104651	0.132353	0.264706	0	− 0.76249
Lasso_alpha_.01	LogisticRegression	0	0.166667	0.633333	0.796078	0.833333	0.7932	0.862903	0.833333	0.266667	1	0.468353
Lasso_alpha_.01	LogisticRegression	1	0.212121	0.627451	0.854902	0.787879	0.767256	0.763424	0.787879	0.333333	0.921569	0.311276
Lasso_alpha_.01	LogisticRegression	2	0.111111	0.809524	0.896825	0.888889	0.88513	0.884848	0.888889	0.666667	0.952381	0.662541
Lasso_alpha_.01	LogisticRegression	3	0.055556	0.904762	0.914683	0.944444	0.943564	0.943622	0.944444	0.833333	0.97619	0.835631
Lasso_alpha_.01	LogisticRegression	4	0.166667	0.714286	0.922619	0.833333	0.824302	0.822222	0.833333	0.5	0.928571	0.478091
Lasso_alpha_.01	LogisticRegression	5	0.018519	0.958333	0.996032	0.981481	0.981188	0.981912	0.981481	0.916667	1	0.946229
Lasso_alpha_.01	LogisticRegression	6	0.203704	0.60119	0.785714	0.796296	0.762192	0.768254	0.796296	0.25	0.952381	0.29027
Lasso_alpha_.01	LogisticRegression	7	0.092593	0.821429	0.829365	0.907407	0.90239	0.906173	0.907407	0.666667	0.97619	0.717137
Lasso_alpha_.01	LogisticRegression	8	0.222222	0.559524	0.686508	0.777778	0.731884	0.733333	0.777778	0.166667	0.952381	0.188982
Lasso_alpha_.01	LogisticRegression	9	0.092593	0.85119	0.894841	0.907407	0.905939	0.905332	0.907407	0.75	0.952381	0.725032
Lasso_alpha_.01	Lasso	Validation	0.867647	0.132353	0.119377	0.132353	0.116883	0.104651	0.132353	0.264706	0	− 0.76249
Lasso_alpha_.01	Lasso	0	0.181818	0.6	0.816993	0.818182	0.767145	0.852814	0.818182	0.2	1	0.402374
Lasso_alpha_.01	Lasso	1	0.227273	0.617647	0.845752	0.772727	0.75531	0.748377	0.772727	0.333333	0.901961	0.27501
Lasso_alpha_.01	Lasso	2	0.12963	0.738095	0.896825	0.87037	0.856955	0.868963	0.87037	0.5	0.97619	0.589384
Lasso_alpha_.01	Lasso	3	0.074074	0.863095	0.912698	0.925926	0.92342	0.924747	0.925926	0.75	0.97619	0.777212
Lasso_alpha_.01	Lasso	4	0.166667	0.714286	0.924603	0.833333	0.824302	0.822222	0.833333	0.5	0.928571	0.478091
Lasso_alpha_.01	Lasso	5	0.055556	0.875	0.996032	0.944444	0.941434	0.948148	0.944444	0.75	1	0.83666
Lasso_alpha_.01	Lasso	6	0.203704	0.60119	0.78373	0.796296	0.762192	0.768254	0.796296	0.25	0.952381	0.29027
Lasso_alpha_.01	Lasso	7	0.148148	0.696429	0.819444	0.851852	0.832099	0.849537	0.851852	0.416667	0.97619	0.519701
Lasso_alpha_.01	Lasso	8	0.203704	0.571429	0.660714	0.796296	0.745042	0.77342	0.796296	0.166667	0.97619	0.259281
Lasso_alpha_.01	Lasso	9	0.092593	0.85119	0.896825	0.907407	0.905939	0.905332	0.907407	0.75	0.952381	0.725032
Lasso_alpha_.01	ElasticNet	validation	0.867647	0.132353	0.117647	0.132353	0.116883	0.104651	0.132353	0.264706	0	− 0.76249
Lasso_alpha_.01	ElasticNet	0	0.181818	0.6	0.815686	0.818182	0.767145	0.852814	0.818182	0.2	1	0.402374
Lasso_alpha_.01	ElasticNet	1	0.227273	0.617647	0.844444	0.772727	0.75531	0.748377	0.772727	0.333333	0.901961	0.27501
Lasso_alpha_.01	ElasticNet	2	0.12963	0.738095	0.896825	0.87037	0.856955	0.868963	0.87037	0.5	0.97619	0.589384
Lasso_alpha_.01	ElasticNet	3	0.074074	0.863095	0.912698	0.925926	0.92342	0.924747	0.925926	0.75	0.97619	0.777212
Lasso_alpha_.01	ElasticNet	4	0.166667	0.714286	0.924603	0.833333	0.824302	0.822222	0.833333	0.5	0.928571	0.478091
Lasso_alpha_.01	ElasticNet	5	0.055556	0.875	0.996032	0.944444	0.941434	0.948148	0.944444	0.75	1	0.83666
Lasso_alpha_.01	ElasticNet	6	0.203704	0.60119	0.785714	0.796296	0.762192	0.768254	0.796296	0.25	0.952381	0.29027
Lasso_alpha_.01	ElasticNet	7	0.148148	0.696429	0.821429	0.851852	0.832099	0.849537	0.851852	0.416667	0.97619	0.519701
Lasso_alpha_.01	ElasticNet	8	0.203704	0.571429	0.660714	0.796296	0.745042	0.77342	0.796296	0.166667	0.97619	0.259281
Lasso_alpha_.01	ElasticNet	9	0.092593	0.85119	0.894841	0.907407	0.905939	0.905332	0.907407	0.75	0.952381	0.725032
Mann_Whitney	RandomForest	Validation	0.088235	0.911765	0.932526	0.911765	0.911458	0.917544	0.911765	0.852941	0.970588	0.829288
Mann_Whitney	RandomForest	0	0.106061	0.813725	0.899346	0.893939	0.889562	0.890572	0.893939	0.666667	0.960784	0.681747
Mann_Whitney	RandomForest	1	0.212121	0.67451	0.69281	0.787879	0.782343	0.778467	0.787879	0.466667	0.882353	0.367765
Mann_Whitney	RandomForest	2	0.148148	0.785714	0.944444	0.851852	0.851852	0.851852	0.851852	0.666667	0.904762	0.571429
Mann_Whitney	RandomForest	3	0.203704	0.720238	0.869048	0.796296	0.799143	0.802585	0.796296	0.583333	0.857143	0.428326
Mann_Whitney	RandomForest	4	0.203704	0.720238	0.861111	0.796296	0.799143	0.802585	0.796296	0.583333	0.857143	0.428326
Mann_Whitney	RandomForest	5	0.185185	0.791667	0.801587	0.814815	0.823413	0.841374	0.814815	0.75	0.833333	0.531105
Mann_Whitney	RandomForest	6	0.425926	0.39881	0.420635	0.574074	0.580027	0.5862	0.574074	0.083333	0.714286	− 0.1968
Mann_Whitney	RandomForest	7	0.203704	0.660714	0.878968	0.796296	0.785258	0.780247	0.796296	0.416667	0.904762	0.358569
Mann_Whitney	RandomForest	8	0.092593	0.880952	0.906746	0.907407	0.908701	0.910778	0.907407	0.833333	0.928571	0.740888
Mann_Whitney	RandomForest	9	0.092593	0.85119	0.962302	0.907407	0.905939	0.905332	0.907407	0.75	0.952381	0.725032
Mann_Whitney	SVC	Validation	0.102941	0.897059	0.943772	0.897059	0.897037	0.897403	0.897059	0.882353	0.911765	0.794461
Mann_Whitney	SVC	0	0.151515	0.807843	0.887582	0.848485	0.851705	0.856706	0.848485	0.733333	0.882353	0.590021
Mann_Whitney	SVC	1	0.227273	0.688235	0.74902	0.772727	0.775268	0.778182	0.772727	0.533333	0.843137	0.368143
Mann_Whitney	SVC	2	0.166667	0.803571	0.911706	0.833333	0.839506	0.851282	0.833333	0.75	0.857143	0.563545
Mann_Whitney	SVC	3	0.203704	0.779762	0.847222	0.796296	0.807411	0.832362	0.796296	0.75	0.809524	0.500851
Mann_Whitney	SVC	4	0.222222	0.708333	0.795635	0.777778	0.783615	0.791667	0.777778	0.583333	0.833333	0.395285
Mann_Whitney	SVC	5	0.166667	0.803571	0.765873	0.833333	0.839506	0.851282	0.833333	0.75	0.857143	0.563545
Mann_Whitney	SVC	6	0.481481	0.363095	0.363095	0.518519	0.540873	0.56652	0.518519	0.083333	0.642857	− 0.24929
Mann_Whitney	SVC	7	0.203704	0.660714	0.763889	0.796296	0.785258	0.780247	0.796296	0.416667	0.904762	0.358569
Mann_Whitney	SVC	8	0.092593	0.910714	0.94246	0.907407	0.910837	0.920798	0.907407	0.916667	0.904762	0.762443
Mann_Whitney	SVC	9	0.111111	0.839286	0.968254	0.888889	0.888889	0.888889	0.888889	0.75	0.928571	0.678571
Mann_Whitney	LogisticRegression	Validation	0.132353	0.867647	0.949827	0.867647	0.865287	0.895349	0.867647	0.735294	1	0.762493
Mann_Whitney	LogisticRegression	0	0.151515	0.690196	0.904575	0.848485	0.826446	0.849659	0.848485	0.4	0.980392	0.517711
Mann_Whitney	LogisticRegression	1	0.181818	0.670588	0.768627	0.818182	0.800505	0.802233	0.818182	0.4	0.941176	0.416631
Mann_Whitney	LogisticRegression	2	0.166667	0.714286	0.914683	0.833333	0.824302	0.822222	0.833333	0.5	0.928571	0.478091
Mann_Whitney	LogisticRegression	3	0.185185	0.613095	0.847222	0.814815	0.77657	0.804444	0.814815	0.25	0.97619	0.359066
Mann_Whitney	LogisticRegression	4	0.203704	0.660714	0.779762	0.796296	0.785258	0.780247	0.796296	0.416667	0.904762	0.358569
Mann_Whitney	LogisticRegression	5	0.203704	0.690476	0.785714	0.796296	0.793066	0.790463	0.796296	0.5	0.880952	0.393238
Mann_Whitney	LogisticRegression	6	0.351852	0.446429	0.386905	0.648148	0.629082	0.612346	0.648148	0.083333	0.809524	− 0.11952
Mann_Whitney	LogisticRegression	7	0.185185	0.613095	0.781746	0.814815	0.77657	0.804444	0.814815	0.25	0.97619	0.359066
Mann_Whitney	LogisticRegression	8	0.185185	0.672619	0.924603	0.814815	0.800505	0.798309	0.814815	0.416667	0.928571	0.404027
Mann_Whitney	LogisticRegression	9	0.111111	0.779762	0.944444	0.888889	0.880303	0.887681	0.888889	0.583333	0.97619	0.654802
Mann_Whitney	Lasso	Validation	0.132353	0.867647	0.949827	0.867647	0.865287	0.895349	0.867647	0.735294	1	0.762493
Mann_Whitney	Lasso	0	0.181818	0.623529	0.89281	0.818182	0.780844	0.815201	0.818182	0.266667	0.980392	0.391274
Mann_Whitney	Lasso	1	0.212121	0.603922	0.781699	0.787879	0.757025	0.75853	0.787879	0.266667	0.941176	0.282873
Mann_Whitney	Lasso	2	0.166667	0.714286	0.93254	0.833333	0.824302	0.822222	0.833333	0.5	0.928571	0.478091
Mann_Whitney	Lasso	3	0.222222	0.529762	0.867063	0.777778	0.710233	0.724359	0.777778	0.083333	0.97619	0.131036
Mann_Whitney	Lasso	4	0.240741	0.577381	0.775794	0.759259	0.734345	0.72408	0.759259	0.25	0.904762	0.19155
Mann_Whitney	Lasso	5	0.222222	0.64881	0.789683	0.777778	0.770261	0.765152	0.777778	0.416667	0.880952	0.318529
Mann_Whitney	Lasso	6	0.259259	0.505952	0.369048	0.740741	0.687198	0.662222	0.740741	0.083333	0.928571	0.018898
Mann_Whitney	Lasso	7	0.166667	0.625	0.789683	0.833333	0.791398	0.862745	0.833333	0.25	1	0.453743
Mann_Whitney	Lasso	8	0.148148	0.72619	0.924603	0.851852	0.840404	0.842995	0.851852	0.5	0.952381	0.529414
Mann_Whitney	Lasso	9	0.111111	0.75	0.926587	0.888889	0.874074	0.902778	0.888889	0.5	1	0.661438
Mann_Whitney	ElasticNet	Validation	0.132353	0.867647	0.949827	0.867647	0.865287	0.895349	0.867647	0.735294	1	0.762493
Mann_Whitney	ElasticNet	0	0.181818	0.623529	0.89281	0.818182	0.780844	0.815201	0.818182	0.266667	0.980392	0.391274
Mann_Whitney	ElasticNet	1	0.19697	0.637255	0.783007	0.80303	0.779381	0.781544	0.80303	0.333333	0.941176	0.352476
Mann_Whitney	ElasticNet	2	0.166667	0.714286	0.93254	0.833333	0.824302	0.822222	0.833333	0.5	0.928571	0.478091
Mann_Whitney	ElasticNet	3	0.222222	0.529762	0.865079	0.777778	0.710233	0.724359	0.777778	0.083333	0.97619	0.131036
Mann_Whitney	ElasticNet	4	0.240741	0.577381	0.771825	0.759259	0.734345	0.72408	0.759259	0.25	0.904762	0.19155
Mann_Whitney	ElasticNet	5	0.222222	0.64881	0.785714	0.777778	0.770261	0.765152	0.777778	0.416667	0.880952	0.318529
Mann_Whitney	ElasticNet	6	0.277778	0.494048	0.365079	0.722222	0.675716	0.647619	0.722222	0.083333	0.904762	− 0.01708
Mann_Whitney	ElasticNet	7	0.166667	0.625	0.785714	0.833333	0.791398	0.862745	0.833333	0.25	1	0.453743
Mann_Whitney	ElasticNet	8	0.148148	0.72619	0.920635	0.851852	0.840404	0.842995	0.851852	0.5	0.952381	0.529414
Mann_Whitney	ElasticNet	9	0.111111	0.75	0.928571	0.888889	0.874074	0.902778	0.888889	0.5	1	0.661438
RandomForest	RandomForest	Validation	0.117647	0.882353	0.932526	0.882353	0.88143	0.894643	0.882353	0.794118	0.970588	0.776899
RandomForest	RandomForest	0	0.106061	0.790196	0.87451	0.893939	0.885811	0.894481	0.893939	0.6	0.980392	0.678357
RandomForest	RandomForest	1	0.19697	0.707843	0.708497	0.80303	0.80059	0.798576	0.80303	0.533333	0.882353	0.426119
RandomForest	RandomForest	2	0.092593	0.85119	0.986111	0.907407	0.905939	0.905332	0.907407	0.75	0.952381	0.725032
RandomForest	RandomForest	3	0.185185	0.791667	0.902778	0.814815	0.823413	0.841374	0.814815	0.75	0.833333	0.531105
RandomForest	RandomForest	4	0.185185	0.732143	0.84127	0.814815	0.814815	0.814815	0.814815	0.583333	0.880952	0.464286
RandomForest	RandomForest	5	0.185185	0.791667	0.882937	0.814815	0.823413	0.841374	0.814815	0.75	0.833333	0.531105
RandomForest	RandomForest	6	0.37037	0.464286	0.484127	0.62963	0.62963	0.62963	0.62963	0.166667	0.761905	− 0.07143
RandomForest	RandomForest	7	0.222222	0.64881	0.863095	0.777778	0.770261	0.765152	0.777778	0.416667	0.880952	0.318529
RandomForest	RandomForest	8	0.148148	0.755952	0.894841	0.851852	0.84684	0.844949	0.851852	0.583333	0.928571	0.547871
RandomForest	RandomForest	9	0.055556	0.904762	0.974206	0.944444	0.943564	0.943622	0.944444	0.833333	0.97619	0.835631
RandomForest	SVC	Validation	0.161765	0.838235	0.923875	0.838235	0.836503	0.853207	0.838235	0.735294	0.941176	0.69128
RandomForest	SVC	0	0.136364	0.770588	0.856209	0.863636	0.858009	0.857323	0.863636	0.6	0.941176	0.588006
RandomForest	SVC	1	0.227273	0.711765	0.816993	0.772727	0.779614	0.789773	0.772727	0.6	0.823529	0.398527
RandomForest	SVC	2	0.074074	0.952381	0.978175	0.925926	0.929365	0.944444	0.925926	1	0.904762	0.823754
RandomForest	SVC	3	0.203704	0.809524	0.880952	0.796296	0.810036	0.850292	0.796296	0.833333	0.785714	0.538925
RandomForest	SVC	4	0.148148	0.815476	0.861111	0.851852	0.855743	0.862302	0.851852	0.75	0.880952	0.598574
RandomForest	SVC	5	0.166667	0.803571	0.843254	0.833333	0.839506	0.851282	0.833333	0.75	0.857143	0.563545
RandomForest	SVC	6	0.388889	0.482143	0.464286	0.611111	0.625514	0.642735	0.611111	0.25	0.714286	− 0.03315
RandomForest	SVC	7	0.259259	0.654762	0.785714	0.740741	0.747551	0.756349	0.740741	0.5	0.809524	0.29364
RandomForest	SVC	8	0.185185	0.702381	0.789683	0.814815	0.808551	0.805051	0.814815	0.5	0.904762	0.4332
RandomForest	SVC	9	0.092593	0.910714	0.97619	0.907407	0.910837	0.920798	0.907407	0.916667	0.904762	0.762443
RandomForest	LogisticRegression	Validation	0.161765	0.838235	0.91609	0.838235	0.833889	0.877778	0.838235	0.676471	1	0.71492
RandomForest	LogisticRegression	0	0.166667	0.633333	0.875817	0.833333	0.7932	0.862903	0.833333	0.266667	1	0.468353
RandomForest	LogisticRegression	1	0.166667	0.727451	0.836601	0.833333	0.826455	0.824074	0.833333	0.533333	0.921569	0.494266
RandomForest	LogisticRegression	2	0.092593	0.821429	0.974206	0.907407	0.90239	0.906173	0.907407	0.666667	0.97619	0.717137
RandomForest	LogisticRegression	3	0.166667	0.654762	0.884921	0.833333	0.80543	0.828571	0.833333	0.333333	0.97619	0.443942
RandomForest	LogisticRegression	4	0.222222	0.619048	0.835317	0.777778	0.760606	0.753623	0.777778	0.333333	0.904762	0.278639
RandomForest	LogisticRegression	5	0.148148	0.785714	0.84127	0.851852	0.851852	0.851852	0.851852	0.666667	0.904762	0.571429
RandomForest	LogisticRegression	6	0.296296	0.482143	0.44246	0.703704	0.664198	0.636574	0.703704	0.083333	0.880952	− 0.04725
RandomForest	LogisticRegression	7	0.12963	0.708333	0.761905	0.87037	0.848668	0.888889	0.87037	0.416667	1	0.597614
RandomForest	LogisticRegression	8	0.185185	0.613095	0.642857	0.814815	0.77657	0.804444	0.814815	0.25	0.97619	0.359066
RandomForest	LogisticRegression	9	0.12963	0.738095	0.984127	0.87037	0.856955	0.868963	0.87037	0.5	0.97619	0.589384
RandomForest	Lasso	Validation	0.161765	0.838235	0.92301	0.838235	0.833889	0.877778	0.838235	0.676471	1	0.71492
RandomForest	Lasso	0	0.181818	0.6	0.860131	0.818182	0.767145	0.852814	0.818182	0.2	1	0.402374
RandomForest	Lasso	1	0.181818	0.670588	0.831373	0.818182	0.800505	0.802233	0.818182	0.4	0.941176	0.416631
RandomForest	Lasso	2	0.12963	0.738095	0.968254	0.87037	0.856955	0.868963	0.87037	0.5	0.97619	0.589384
RandomForest	Lasso	3	0.222222	0.529762	0.894841	0.777778	0.710233	0.724359	0.777778	0.083333	0.97619	0.131036
RandomForest	Lasso	4	0.222222	0.589286	0.809524	0.777778	0.748148	0.743056	0.777778	0.25	0.928571	0.236228
RandomForest	Lasso	5	0.185185	0.702381	0.815476	0.814815	0.808551	0.805051	0.814815	0.5	0.904762	0.4332
RandomForest	Lasso	6	0.259259	0.505952	0.446429	0.740741	0.687198	0.662222	0.740741	0.083333	0.928571	0.018898
RandomForest	Lasso	7	0.148148	0.666667	0.746032	0.851852	0.821256	0.875556	0.851852	0.333333	1	0.52915
RandomForest	Lasso	8	0.185185	0.613095	0.704365	0.814815	0.77657	0.804444	0.814815	0.25	0.97619	0.359066
RandomForest	Lasso	9	0.111111	0.75	0.980159	0.888889	0.874074	0.902778	0.888889	0.5	1	0.661438
RandomForest	ElasticNet	Validation	0.161765	0.838235	0.922145	0.838235	0.833889	0.877778	0.838235	0.676471	1	0.71492
RandomForest	ElasticNet	0	0.181818	0.6	0.861438	0.818182	0.767145	0.852814	0.818182	0.2	1	0.402374
RandomForest	ElasticNet	1	0.181818	0.670588	0.835294	0.818182	0.800505	0.802233	0.818182	0.4	0.941176	0.416631
RandomForest	ElasticNet	2	0.12963	0.738095	0.968254	0.87037	0.856955	0.868963	0.87037	0.5	0.97619	0.589384
RandomForest	ElasticNet	3	0.222222	0.529762	0.894841	0.777778	0.710233	0.724359	0.777778	0.083333	0.97619	0.131036
RandomForest	ElasticNet	4	0.222222	0.589286	0.809524	0.777778	0.748148	0.743056	0.777778	0.25	0.928571	0.236228
RandomForest	ElasticNet	5	0.185185	0.702381	0.815476	0.814815	0.808551	0.805051	0.814815	0.5	0.904762	0.4332
RandomForest	ElasticNet	6	0.240741	0.517857	0.446429	0.759259	0.698686	0.684096	0.759259	0.083333	0.952381	0.06482
RandomForest	ElasticNet	7	0.148148	0.666667	0.746032	0.851852	0.821256	0.875556	0.851852	0.333333	1	0.52915
RandomForest	ElasticNet	8	0.185185	0.613095	0.686508	0.814815	0.77657	0.804444	0.814815	0.25	0.97619	0.359066
RandomForest	ElasticNet	9	0.111111	0.75	0.982143	0.888889	0.874074	0.902778	0.888889	0.5	1	0.661438
RFE_RF	RandomForest	Validation	0.102941	0.897059	0.933391	0.897059	0.895956	0.914634	0.897059	0.794118	1	0.811503
RFE_RF	RandomForest	0	0.090909	0.823529	0.89281	0.909091	0.903813	0.909091	0.909091	0.666667	0.980392	0.727607
RFE_RF	RandomForest	1	0.212121	0.67451	0.861438	0.787879	0.782343	0.778467	0.787879	0.466667	0.882353	0.367765
RFE_RF	RandomForest	2	0.111111	0.869048	0.952381	0.888889	0.891807	0.897619	0.888889	0.833333	0.904762	0.700219
RFE_RF	RandomForest	3	0.12963	0.857143	0.936508	0.87037	0.875171	0.88604	0.87037	0.833333	0.880952	0.662994
RFE_RF	RandomForest	4	0.148148	0.815476	0.863095	0.851852	0.855743	0.862302	0.851852	0.75	0.880952	0.598574
RFE_RF	RandomForest	5	0.185185	0.791667	0.875	0.814815	0.823413	0.841374	0.814815	0.75	0.833333	0.531105
RFE_RF	RandomForest	6	0.407407	0.410714	0.611111	0.592593	0.592593	0.592593	0.592593	0.083333	0.738095	− 0.17857
RFE_RF	RandomForest	7	0.203704	0.660714	0.857143	0.796296	0.785258	0.780247	0.796296	0.416667	0.904762	0.358569
RFE_RF	RandomForest	8	0.111111	0.839286	0.869048	0.888889	0.888889	0.888889	0.888889	0.75	0.928571	0.678571
RFE_RF	RandomForest	9	0.074074	0.922619	0.956349	0.925926	0.927872	0.932937	0.925926	0.916667	0.928571	0.801863
RFE_RF	SVC	Validation	0.117647	0.882353	0.947232	0.882353	0.882251	0.883681	0.882353	0.852941	0.911765	0.766032
RFE_RF	SVC	0	0.166667	0.727451	0.878431	0.833333	0.826455	0.824074	0.833333	0.533333	0.921569	0.494266
RFE_RF	SVC	1	0.19697	0.731373	0.870588	0.80303	0.805232	0.807841	0.80303	0.6	0.862745	0.452509
RFE_RF	SVC	2	0.092593	0.910714	0.968254	0.907407	0.910837	0.920798	0.907407	0.916667	0.904762	0.762443
RFE_RF	SVC	3	0.185185	0.821429	0.902778	0.814815	0.826211	0.858025	0.814815	0.833333	0.809524	0.566947
RFE_RF	SVC	4	0.111111	0.89881	0.894841	0.888889	0.894048	0.910088	0.888889	0.916667	0.880952	0.726205
RFE_RF	SVC	5	0.203704	0.779762	0.867063	0.796296	0.807411	0.832362	0.796296	0.75	0.809524	0.500851
RFE_RF	SVC	6	0.388889	0.452381	0.460317	0.611111	0.616546	0.622264	0.611111	0.166667	0.738095	− 0.09261
RFE_RF	SVC	7	0.185185	0.732143	0.793651	0.814815	0.814815	0.814815	0.814815	0.583333	0.880952	0.464286
RFE_RF	SVC	8	0.185185	0.702381	0.809524	0.814815	0.808551	0.805051	0.814815	0.5	0.904762	0.4332
RFE_RF	SVC	9	0.148148	0.815476	0.890873	0.851852	0.855743	0.862302	0.851852	0.75	0.880952	0.598574
RFE_RF	LogisticRegression	Validation	0.161765	0.838235	0.944637	0.838235	0.835351	0.863721	0.838235	0.705882	0.970588	0.701493
RFE_RF	LogisticRegression	0	0.166667	0.633333	0.890196	0.833333	0.7932	0.862903	0.833333	0.266667	1	0.468353
RFE_RF	LogisticRegression	1	0.166667	0.703922	0.861438	0.833333	0.820561	0.821429	0.833333	0.466667	0.941176	0.476683
RFE_RF	LogisticRegression	2	0.111111	0.75	0.97619	0.888889	0.874074	0.902778	0.888889	0.5	1	0.661438
RFE_RF	LogisticRegression	3	0.074074	0.863095	0.888889	0.925926	0.92342	0.924747	0.925926	0.75	0.97619	0.777212
RFE_RF	LogisticRegression	4	0.166667	0.714286	0.894841	0.833333	0.824302	0.822222	0.833333	0.5	0.928571	0.478091
RFE_RF	LogisticRegression	5	0.148148	0.815476	0.833333	0.851852	0.855743	0.862302	0.851852	0.75	0.880952	0.598574
RFE_RF	LogisticRegression	6	0.277778	0.52381	0.470238	0.722222	0.693475	0.675785	0.722222	0.166667	0.880952	0.058938
RFE_RF	LogisticRegression	7	0.166667	0.684524	0.751984	0.833333	0.816085	0.820669	0.833333	0.416667	0.952381	0.456772
RFE_RF	LogisticRegression	8	0.277778	0.464286	0.728175	0.722222	0.65233	0.594771	0.722222	0	0.928571	− 0.12964
RFE_RF	LogisticRegression	9	0.166667	0.714286	0.875	0.833333	0.824302	0.822222	0.833333	0.5	0.928571	0.478091
RFE_RF	Lasso	Validation	0.176471	0.823529	0.943772	0.823529	0.819629	0.854167	0.823529	0.676471	0.970588	0.677003
RFE_RF	Lasso	0	0.166667	0.633333	0.870588	0.833333	0.7932	0.862903	0.833333	0.266667	1	0.468353
RFE_RF	Lasso	1	0.166667	0.703922	0.867974	0.833333	0.820561	0.821429	0.833333	0.466667	0.941176	0.476683
RFE_RF	Lasso	2	0.111111	0.75	0.96627	0.888889	0.874074	0.902778	0.888889	0.5	1	0.661438
RFE_RF	Lasso	3	0.111111	0.779762	0.896825	0.888889	0.880303	0.887681	0.888889	0.583333	0.97619	0.654802
RFE_RF	Lasso	4	0.185185	0.642857	0.896825	0.814815	0.790123	0.796296	0.814815	0.333333	0.952381	0.377964
RFE_RF	Lasso	5	0.148148	0.785714	0.84127	0.851852	0.851852	0.851852	0.851852	0.666667	0.904762	0.571429
RFE_RF	Lasso	6	0.240741	0.517857	0.494048	0.759259	0.698686	0.684096	0.759259	0.083333	0.952381	0.06482
RFE_RF	Lasso	7	0.185185	0.642857	0.761905	0.814815	0.790123	0.796296	0.814815	0.333333	0.952381	0.377964
RFE_RF	Lasso	8	0.277778	0.464286	0.769841	0.722222	0.65233	0.594771	0.722222	0	0.928571	− 0.12964
RFE_RF	Lasso	9	0.185185	0.672619	0.878968	0.814815	0.800505	0.798309	0.814815	0.416667	0.928571	0.404027
RFE_RF	ElasticNet	Validation	0.176471	0.823529	0.942042	0.823529	0.819629	0.854167	0.823529	0.676471	0.970588	0.677003
RFE_RF	ElasticNet	0	0.166667	0.633333	0.870588	0.833333	0.7932	0.862903	0.833333	0.266667	1	0.468353
RFE_RF	ElasticNet	1	0.166667	0.703922	0.869281	0.833333	0.820561	0.821429	0.833333	0.466667	0.941176	0.476683
RFE_RF	ElasticNet	2	0.111111	0.75	0.96627	0.888889	0.874074	0.902778	0.888889	0.5	1	0.661438
RFE_RF	ElasticNet	3	0.111111	0.779762	0.890873	0.888889	0.880303	0.887681	0.888889	0.583333	0.97619	0.654802
RFE_RF	ElasticNet	4	0.185185	0.642857	0.894841	0.814815	0.790123	0.796296	0.814815	0.333333	0.952381	0.377964
RFE_RF	ElasticNet	5	0.148148	0.785714	0.845238	0.851852	0.851852	0.851852	0.851852	0.666667	0.904762	0.571429
RFE_RF	ElasticNet	6	0.240741	0.517857	0.496032	0.759259	0.698686	0.684096	0.759259	0.083333	0.952381	0.06482
RFE_RF	ElasticNet	7	0.185185	0.642857	0.761905	0.814815	0.790123	0.796296	0.814815	0.333333	0.952381	0.377964
RFE_RF	ElasticNet	8	0.277778	0.464286	0.757937	0.722222	0.65233	0.594771	0.722222	0	0.928571	− 0.12964
RFE_RF	ElasticNet	9	0.185185	0.672619	0.880952	0.814815	0.800505	0.798309	0.814815	0.416667	0.928571	0.404027
RFE_SVM	RandomForest	Validation	0.235294	0.764706	0.817474	0.764706	0.761404	0.78022	0.764706	0.647059	0.882353	0.544705
RFE_SVM	RandomForest	0	0.227273	0.547059	0.750327	0.772727	0.718	0.72434	0.772727	0.133333	0.960784	0.165301
RFE_SVM	RandomForest	1	0.272727	0.682353	0.801307	0.727273	0.741477	0.7671	0.727273	0.6	0.764706	0.328139
RFE_SVM	RandomForest	2	0.037037	0.916667	0.992063	0.962963	0.96171	0.964646	0.962963	0.833333	1	0.891883
RFE_SVM	RandomForest	3	0.185185	0.761905	0.865079	0.814815	0.819679	0.826984	0.814815	0.666667	0.857143	0.496929
RFE_SVM	RandomForest	4	0.092593	0.910714	0.928571	0.907407	0.910837	0.920798	0.907407	0.916667	0.904762	0.762443
RFE_SVM	RandomForest	5	0.148148	0.785714	0.900794	0.851852	0.851852	0.851852	0.851852	0.666667	0.904762	0.571429
RFE_SVM	RandomForest	6	0.203704	0.571429	0.710317	0.796296	0.745042	0.77342	0.796296	0.166667	0.97619	0.259281
RFE_SVM	RandomForest	7	0.148148	0.755952	0.894841	0.851852	0.84684	0.844949	0.851852	0.583333	0.928571	0.547871
RFE_SVM	RandomForest	8	0.222222	0.619048	0.845238	0.777778	0.760606	0.753623	0.777778	0.333333	0.904762	0.278639
RFE_SVM	RandomForest	9	0.092593	0.85119	0.831349	0.907407	0.905939	0.905332	0.907407	0.75	0.952381	0.725032
RFE_SVM	SVC	Validation	0.279412	0.720588	0.865052	0.720588	0.696927	0.820755	0.720588	0.441176	1	0.531995
RFE_SVM	SVC	0	0.212121	0.792157	0.845752	0.787879	0.801181	0.837393	0.787879	0.8	0.784314	0.5139
RFE_SVM	SVC	1	0.181818	0.835294	0.894118	0.818182	0.829584	0.865245	0.818182	0.866667	0.803922	0.589778
RFE_SVM	SVC	2	0.148148	0.785714	0.956349	0.851852	0.851852	0.851852	0.851852	0.666667	0.904762	0.571429
RFE_SVM	SVC	3	0.185185	0.880952	0.962302	0.814815	0.829535	0.89899	0.814815	1	0.761905	0.644658
RFE_SVM	SVC	4	0.074074	0.922619	0.956349	0.925926	0.927872	0.932937	0.925926	0.916667	0.928571	0.801863
RFE_SVM	SVC	5	0.111111	0.928571	0.956349	0.888889	0.895726	0.925926	0.888889	1	0.857143	0.755929
RFE_SVM	SVC	6	0.222222	0.64881	0.738095	0.777778	0.770261	0.765152	0.777778	0.416667	0.880952	0.318529
RFE_SVM	SVC	7	0.055556	0.934524	0.968254	0.944444	0.945221	0.946842	0.944444	0.916667	0.952381	0.845075
RFE_SVM	SVC	8	0.12963	0.827381	0.944444	0.87037	0.872182	0.874713	0.87037	0.75	0.904762	0.6367
RFE_SVM	SVC	9	0.148148	0.875	0.918651	0.851852	0.860969	0.891975	0.851852	0.916667	0.833333	0.661438
RFE_SVM	LogisticRegression	Validation	0.323529	0.676471	0.845156	0.676471	0.638647	0.803571	0.676471	0.352941	1	0.46291
RFE_SVM	LogisticRegression	0	0.212121	0.603922	0.854902	0.787879	0.757025	0.75853	0.787879	0.266667	0.941176	0.282873
RFE_SVM	LogisticRegression	1	0.166667	0.77451	0.901961	0.833333	0.835196	0.8375	0.833333	0.666667	0.882353	0.536875
RFE_SVM	LogisticRegression	2	0.074074	0.833333	0.952381	0.925926	0.920202	0.932367	0.925926	0.666667	1	0.780189
RFE_SVM	LogisticRegression	3	0.092593	0.791667	0.964286	0.907407	0.897825	0.917258	0.907407	0.583333	1	0.721995
RFE_SVM	LogisticRegression	4	0.092593	0.880952	0.964286	0.907407	0.908701	0.910778	0.907407	0.833333	0.928571	0.740888
RFE_SVM	LogisticRegression	5	0.111111	0.839286	0.950397	0.888889	0.888889	0.888889	0.888889	0.75	0.928571	0.678571
RFE_SVM	LogisticRegression	6	0.222222	0.529762	0.730159	0.777778	0.710233	0.724359	0.777778	0.083333	0.97619	0.131036
RFE_SVM	LogisticRegression	7	0.092593	0.791667	0.946429	0.907407	0.897825	0.917258	0.907407	0.583333	1	0.721995
RFE_SVM	LogisticRegression	8	0.092593	0.791667	0.910714	0.907407	0.897825	0.917258	0.907407	0.583333	1	0.721995
RFE_SVM	LogisticRegression	9	0.166667	0.744048	0.886905	0.833333	0.830691	0.828753	0.833333	0.583333	0.904762	0.503836
RFE_SVM	Lasso	Validation	0.323529	0.676471	0.849481	0.676471	0.638647	0.803571	0.676471	0.352941	1	0.46291
RFE_SVM	Lasso	0	0.181818	0.623529	0.847059	0.818182	0.780844	0.815201	0.818182	0.266667	0.980392	0.391274
RFE_SVM	Lasso	1	0.19697	0.684314	0.896732	0.80303	0.794901	0.790825	0.80303	0.466667	0.901961	0.400526
RFE_SVM	Lasso	2	0.074074	0.833333	0.96627	0.925926	0.920202	0.932367	0.925926	0.666667	1	0.780189
RFE_SVM	Lasso	3	0.092593	0.791667	0.946429	0.907407	0.897825	0.917258	0.907407	0.583333	1	0.721995
RFE_SVM	Lasso	4	0.092593	0.85119	0.96627	0.907407	0.905939	0.905332	0.907407	0.75	0.952381	0.725032
RFE_SVM	Lasso	5	0.111111	0.809524	0.952381	0.888889	0.88513	0.884848	0.888889	0.666667	0.952381	0.662541
RFE_SVM	Lasso	6	0.222222	0.529762	0.742063	0.777778	0.710233	0.724359	0.777778	0.083333	0.97619	0.131036
RFE_SVM	Lasso	7	0.12963	0.708333	0.952381	0.87037	0.848668	0.888889	0.87037	0.416667	1	0.597614
RFE_SVM	Lasso	8	0.092593	0.791667	0.924603	0.907407	0.897825	0.917258	0.907407	0.583333	1	0.721995
RFE_SVM	Lasso	9	0.148148	0.755952	0.90873	0.851852	0.84684	0.844949	0.851852	0.583333	0.928571	0.547871
RFE_SVM	ElasticNet	Validation	0.323529	0.676471	0.851211	0.676471	0.638647	0.803571	0.676471	0.352941	1	0.46291
RFE_SVM	ElasticNet	0	0.181818	0.623529	0.844444	0.818182	0.780844	0.815201	0.818182	0.266667	0.980392	0.391274
RFE_SVM	ElasticNet	1	0.19697	0.684314	0.895425	0.80303	0.794901	0.790825	0.80303	0.466667	0.901961	0.400526
RFE_SVM	ElasticNet	2	0.074074	0.833333	0.96627	0.925926	0.920202	0.932367	0.925926	0.666667	1	0.780189
RFE_SVM	ElasticNet	3	0.092593	0.791667	0.94246	0.907407	0.897825	0.917258	0.907407	0.583333	1	0.721995
RFE_SVM	ElasticNet	4	0.111111	0.839286	0.96627	0.888889	0.888889	0.888889	0.888889	0.75	0.928571	0.678571
RFE_SVM	ElasticNet	5	0.12963	0.797619	0.954365	0.87037	0.868315	0.867043	0.87037	0.666667	0.928571	0.614434
RFE_SVM	ElasticNet	6	0.222222	0.529762	0.748016	0.777778	0.710233	0.724359	0.777778	0.083333	0.97619	0.131036
RFE_SVM	ElasticNet	7	0.12963	0.708333	0.956349	0.87037	0.848668	0.888889	0.87037	0.416667	1	0.597614
RFE_SVM	ElasticNet	8	0.092593	0.791667	0.926587	0.907407	0.897825	0.917258	0.907407	0.583333	1	0.721995
RFE_SVM	ElasticNet	9	0.148148	0.755952	0.90873	0.851852	0.84684	0.844949	0.851852	0.583333	0.928571	0.547871
RidgeCV	RandomForest	Validation	0.235294	0.764706	0.82699	0.764706	0.763889	0.768421	0.764706	0.705882	0.823529	0.533114
RidgeCV	RandomForest	0	0.19697	0.590196	0.785621	0.80303	0.7556	0.793622	0.80303	0.2	0.980392	0.316827
RidgeCV	RandomForest	1	0.181818	0.694118	0.747712	0.818182	0.807626	0.804959	0.818182	0.466667	0.921569	0.436564
RidgeCV	RandomForest	2	0.203704	0.660714	0.799603	0.796296	0.785258	0.780247	0.796296	0.416667	0.904762	0.358569
RidgeCV	RandomForest	3	0.222222	0.64881	0.789683	0.777778	0.770261	0.765152	0.777778	0.416667	0.880952	0.318529
RidgeCV	RandomForest	4	0.222222	0.767857	0.825397	0.777778	0.791453	0.824074	0.777778	0.75	0.785714	0.472456
RidgeCV	RandomForest	5	0.166667	0.744048	0.827381	0.833333	0.830691	0.828753	0.833333	0.583333	0.904762	0.503836
RidgeCV	RandomForest	6	0.240741	0.607143	0.672619	0.759259	0.746214	0.738272	0.759259	0.333333	0.880952	0.239046
RidgeCV	RandomForest	7	0.148148	0.785714	0.845238	0.851852	0.851852	0.851852	0.851852	0.666667	0.904762	0.571429
RidgeCV	RandomForest	8	0.240741	0.577381	0.704365	0.759259	0.734345	0.72408	0.759259	0.25	0.904762	0.19155
RidgeCV	RandomForest	9	0.092593	0.85119	0.93254	0.907407	0.905939	0.905332	0.907407	0.75	0.952381	0.725032
RidgeCV	SVC	Validation	0.279412	0.720588	0.874567	0.720588	0.709989	0.758359	0.720588	0.529412	0.911765	0.477455
RidgeCV	SVC	0	0.151515	0.807843	0.871895	0.848485	0.851705	0.856706	0.848485	0.733333	0.882353	0.590021
RidgeCV	SVC	1	0.30303	0.662745	0.775163	0.69697	0.715973	0.753838	0.69697	0.6	0.72549	0.286266
RidgeCV	SVC	2	0.222222	0.767857	0.837302	0.777778	0.791453	0.824074	0.777778	0.75	0.785714	0.472456
RidgeCV	SVC	3	0.259259	0.654762	0.744048	0.740741	0.747551	0.756349	0.740741	0.5	0.809524	0.29364
RidgeCV	SVC	4	0.351852	0.684524	0.763889	0.648148	0.677748	0.777318	0.648148	0.75	0.619048	0.307701
RidgeCV	SVC	5	0.166667	0.833333	0.892857	0.833333	0.842427	0.866455	0.833333	0.833333	0.833333	0.596759
RidgeCV	SVC	6	0.333333	0.577381	0.634921	0.666667	0.682143	0.703947	0.666667	0.416667	0.738095	0.140905
RidgeCV	SVC	7	0.222222	0.767857	0.835317	0.777778	0.791453	0.824074	0.777778	0.75	0.785714	0.472456
RidgeCV	SVC	8	0.148148	0.785714	0.813492	0.851852	0.851852	0.851852	0.851852	0.666667	0.904762	0.571429
RidgeCV	SVC	9	0.259259	0.744048	0.894841	0.740741	0.759503	0.80915	0.740741	0.75	0.738095	0.420209
RidgeCV	LogisticRegression	Validation	0.397059	0.602941	0.865917	0.602941	0.540885	0.724105	0.602941	0.235294	0.970588	0.303774
RidgeCV	LogisticRegression	0	0.212121	0.580392	0.873203	0.787879	0.744318	0.757079	0.787879	0.2	0.960784	0.254639
RidgeCV	LogisticRegression	1	0.151515	0.690196	0.772549	0.848485	0.826446	0.849659	0.848485	0.4	0.980392	0.517711
RidgeCV	LogisticRegression	2	0.185185	0.583333	0.849206	0.814815	0.758528	0.850427	0.814815	0.166667	1	0.3669
RidgeCV	LogisticRegression	3	0.185185	0.613095	0.763889	0.814815	0.77657	0.804444	0.814815	0.25	0.97619	0.359066
RidgeCV	LogisticRegression	4	0.240741	0.755952	0.765873	0.759259	0.775497	0.816374	0.759259	0.75	0.761905	0.44565
RidgeCV	LogisticRegression	5	0.148148	0.72619	0.882937	0.851852	0.840404	0.842995	0.851852	0.5	0.952381	0.529414
RidgeCV	LogisticRegression	6	0.277778	0.52381	0.628968	0.722222	0.693475	0.675785	0.722222	0.166667	0.880952	0.058938
RidgeCV	LogisticRegression	7	0.203704	0.571429	0.833333	0.796296	0.745042	0.77342	0.796296	0.166667	0.97619	0.259281
RidgeCV	LogisticRegression	8	0.203704	0.60119	0.738095	0.796296	0.762192	0.768254	0.796296	0.25	0.952381	0.29027
RidgeCV	LogisticRegression	9	0.148148	0.72619	0.902778	0.851852	0.840404	0.842995	0.851852	0.5	0.952381	0.529414
RidgeCV	Lasso	Validation	0.367647	0.632353	0.885813	0.632353	0.58486	0.744019	0.632353	0.294118	0.970588	0.359425
RidgeCV	Lasso	0	0.227273	0.523529	0.870588	0.772727	0.698675	0.71733	0.772727	0.066667	0.980392	0.115045
RidgeCV	Lasso	1	0.181818	0.623529	0.760784	0.818182	0.780844	0.815201	0.818182	0.266667	0.980392	0.391274
RidgeCV	Lasso	2	0.203704	0.541667	0.857143	0.796296	0.721907	0.838574	0.796296	0.083333	1	0.256978
RidgeCV	Lasso	3	0.222222	0.529762	0.779762	0.777778	0.710233	0.724359	0.777778	0.083333	0.97619	0.131036
RidgeCV	Lasso	4	0.166667	0.803571	0.757937	0.833333	0.839506	0.851282	0.833333	0.75	0.857143	0.563545
RidgeCV	Lasso	5	0.12963	0.738095	0.894841	0.87037	0.856955	0.868963	0.87037	0.5	0.97619	0.589384
RidgeCV	Lasso	6	0.240741	0.547619	0.640873	0.759259	0.718954	0.707937	0.759259	0.166667	0.928571	0.136598
RidgeCV	Lasso	7	0.203704	0.541667	0.837302	0.796296	0.721907	0.838574	0.796296	0.083333	1	0.256978
RidgeCV	Lasso	8	0.203704	0.541667	0.77381	0.796296	0.721907	0.838574	0.796296	0.083333	1	0.256978
RidgeCV	Lasso	9	0.148148	0.72619	0.906746	0.851852	0.840404	0.842995	0.851852	0.5	0.952381	0.529414
RidgeCV	ElasticNet	Validation	0.382353	0.617647	0.884948	0.617647	0.563241	0.734483	0.617647	0.264706	0.970588	0.332182
RidgeCV	ElasticNet	0	0.227273	0.523529	0.873203	0.772727	0.698675	0.71733	0.772727	0.066667	0.980392	0.115045
RidgeCV	ElasticNet	1	0.181818	0.623529	0.762092	0.818182	0.780844	0.815201	0.818182	0.266667	0.980392	0.391274
RidgeCV	ElasticNet	2	0.203704	0.541667	0.855159	0.796296	0.721907	0.838574	0.796296	0.083333	1	0.256978
RidgeCV	ElasticNet	3	0.222222	0.529762	0.779762	0.777778	0.710233	0.724359	0.777778	0.083333	0.97619	0.131036
RidgeCV	ElasticNet	4	0.166667	0.803571	0.757937	0.833333	0.839506	0.851282	0.833333	0.75	0.857143	0.563545
RidgeCV	ElasticNet	5	0.12963	0.738095	0.894841	0.87037	0.856955	0.868963	0.87037	0.5	0.97619	0.589384
RidgeCV	ElasticNet	6	0.240741	0.547619	0.638889	0.759259	0.718954	0.707937	0.759259	0.166667	0.928571	0.136598
RidgeCV	ElasticNet	7	0.203704	0.541667	0.833333	0.796296	0.721907	0.838574	0.796296	0.083333	1	0.256978
RidgeCV	ElasticNet	8	0.203704	0.541667	0.77381	0.796296	0.721907	0.838574	0.796296	0.083333	1	0.256978
RidgeCV	ElasticNet	9	0.148148	0.72619	0.906746	0.851852	0.840404	0.842995	0.851852	0.5	0.952381	0.529414
SVM	RandomForest	Validation	0.308824	0.691176	0.831315	0.691176	0.658618	0.809091	0.691176	0.382353	1	0.486172
SVM	RandomForest	0	0.19697	0.566667	0.721569	0.80303	0.738851	0.84304	0.80303	0.133333	1	0.32596
SVM	RandomForest	1	0.257576	0.598039	0.730719	0.742424	0.731794	0.724327	0.742424	0.333333	0.862745	0.213046
SVM	RandomForest	2	0.222222	0.559524	0.642857	0.777778	0.731884	0.733333	0.777778	0.166667	0.952381	0.188982
SVM	RandomForest	3	0.185185	0.702381	0.84127	0.814815	0.808551	0.805051	0.814815	0.5	0.904762	0.4332
SVM	RandomForest	4	0.222222	0.738095	0.819444	0.777778	0.788095	0.807018	0.777778	0.666667	0.809524	0.433555
SVM	RandomForest	5	0.166667	0.744048	0.759921	0.833333	0.830691	0.828753	0.833333	0.583333	0.904762	0.503836
SVM	RandomForest	6	0.222222	0.64881	0.755952	0.777778	0.770261	0.765152	0.777778	0.416667	0.880952	0.318529
SVM	RandomForest	7	0.240741	0.607143	0.740079	0.759259	0.746214	0.738272	0.759259	0.333333	0.880952	0.239046
SVM	RandomForest	8	0.240741	0.547619	0.77381	0.759259	0.718954	0.707937	0.759259	0.166667	0.928571	0.136598
SVM	RandomForest	9	0.148148	0.72619	0.855159	0.851852	0.840404	0.842995	0.851852	0.5	0.952381	0.529414
SVM	SVC	Validation	0.294118	0.705882	0.776817	0.705882	0.704861	0.708772	0.705882	0.647059	0.764706	0.414644
SVM	SVC	0	0.212121	0.721569	0.814379	0.787879	0.792386	0.798428	0.787879	0.6	0.843137	0.424665
SVM	SVC	1	0.212121	0.721569	0.752941	0.787879	0.792386	0.798428	0.787879	0.6	0.843137	0.424665
SVM	SVC	2	0.277778	0.672619	0.706349	0.722222	0.737378	0.764176	0.722222	0.583333	0.761905	0.309036
SVM	SVC	3	0.222222	0.738095	0.843254	0.777778	0.788095	0.807018	0.777778	0.666667	0.809524	0.433555
SVM	SVC	4	0.333333	0.666667	0.819444	0.666667	0.693164	0.761364	0.666667	0.666667	0.666667	0.282038
SVM	SVC	5	0.259259	0.714286	0.825397	0.740741	0.756695	0.790123	0.740741	0.666667	0.761905	0.377964
SVM	SVC	6	0.37037	0.642857	0.751984	0.62963	0.660494	0.748148	0.62963	0.666667	0.619048	0.239046
SVM	SVC	7	0.314815	0.589286	0.636905	0.685185	0.696845	0.712251	0.685185	0.416667	0.761905	0.165748
SVM	SVC	8	0.277778	0.672619	0.835317	0.722222	0.737378	0.764176	0.722222	0.583333	0.761905	0.309036
SVM	SVC	9	0.314815	0.678571	0.789683	0.685185	0.709226	0.768158	0.685185	0.666667	0.690476	0.304572
SVM	LogisticRegression	Validation	0.264706	0.735294	0.760381	0.735294	0.71978	0.802222	0.735294	0.5	0.970588	0.533333
SVM	LogisticRegression	0	0.227273	0.523529	0.786928	0.772727	0.698675	0.71733	0.772727	0.066667	0.980392	0.115045
SVM	LogisticRegression	1	0.212121	0.580392	0.743791	0.787879	0.744318	0.757079	0.787879	0.2	0.960784	0.254639
SVM	LogisticRegression	2	0.185185	0.583333	0.698413	0.814815	0.758528	0.850427	0.814815	0.166667	1	0.3669
SVM	LogisticRegression	3	0.148148	0.666667	0.835317	0.851852	0.821256	0.875556	0.851852	0.333333	1	0.52915
SVM	LogisticRegression	4	0.203704	0.720238	0.827381	0.796296	0.799143	0.802585	0.796296	0.583333	0.857143	0.428326
SVM	LogisticRegression	5	0.12963	0.738095	0.823413	0.87037	0.856955	0.868963	0.87037	0.5	0.97619	0.589384
SVM	LogisticRegression	6	0.259259	0.505952	0.75	0.740741	0.687198	0.662222	0.740741	0.083333	0.928571	0.018898
SVM	LogisticRegression	7	0.240741	0.547619	0.625	0.759259	0.718954	0.707937	0.759259	0.166667	0.928571	0.136598
SVM	LogisticRegression	8	0.166667	0.625	0.805556	0.833333	0.791398	0.862745	0.833333	0.25	1	0.453743
SVM	LogisticRegression	9	0.111111	0.75	0.746032	0.888889	0.874074	0.902778	0.888889	0.5	1	0.661438
SVM	Lasso	Validation	0.323529	0.676471	0.763841	0.676471	0.645833	0.769841	0.676471	0.382353	0.970588	0.436436
SVM	Lasso	0	0.212121	0.533333	0.789542	0.787879	0.707876	0.833566	0.787879	0.066667	1	0.228709
SVM	Lasso	1	0.212121	0.580392	0.741176	0.787879	0.744318	0.757079	0.787879	0.2	0.960784	0.254639
SVM	Lasso	2	0.203704	0.541667	0.68254	0.796296	0.721907	0.838574	0.796296	0.083333	1	0.256978
SVM	Lasso	3	0.185185	0.583333	0.837302	0.814815	0.758528	0.850427	0.814815	0.166667	1	0.3669
SVM	Lasso	4	0.148148	0.755952	0.819444	0.851852	0.84684	0.844949	0.851852	0.583333	0.928571	0.547871
SVM	Lasso	5	0.12963	0.708333	0.825397	0.87037	0.848668	0.888889	0.87037	0.416667	1	0.597614
SVM	Lasso	6	0.222222	0.529762	0.759921	0.777778	0.710233	0.724359	0.777778	0.083333	0.97619	0.131036
SVM	Lasso	7	0.203704	0.571429	0.625	0.796296	0.745042	0.77342	0.796296	0.166667	0.97619	0.259281
SVM	Lasso	8	0.185185	0.583333	0.823413	0.814815	0.758528	0.850427	0.814815	0.166667	1	0.3669
SVM	Lasso	9	0.111111	0.75	0.791667	0.888889	0.874074	0.902778	0.888889	0.5	1	0.661438
SVM	ElasticNet	Validation	0.323529	0.676471	0.763841	0.676471	0.645833	0.769841	0.676471	0.382353	0.970588	0.436436
SVM	ElasticNet	0	0.212121	0.533333	0.789542	0.787879	0.707876	0.833566	0.787879	0.066667	1	0.228709
SVM	ElasticNet	1	0.212121	0.580392	0.741176	0.787879	0.744318	0.757079	0.787879	0.2	0.960784	0.254639
SVM	ElasticNet	2	0.203704	0.541667	0.68254	0.796296	0.721907	0.838574	0.796296	0.083333	1	0.256978
SVM	ElasticNet	3	0.185185	0.583333	0.835317	0.814815	0.758528	0.850427	0.814815	0.166667	1	0.3669
SVM	ElasticNet	4	0.148148	0.755952	0.819444	0.851852	0.84684	0.844949	0.851852	0.583333	0.928571	0.547871
SVM	ElasticNet	5	0.12963	0.708333	0.831349	0.87037	0.848668	0.888889	0.87037	0.416667	1	0.597614
SVM	ElasticNet	6	0.222222	0.529762	0.761905	0.777778	0.710233	0.724359	0.777778	0.083333	0.97619	0.131036
SVM	ElasticNet	7	0.203704	0.571429	0.626984	0.796296	0.745042	0.77342	0.796296	0.166667	0.97619	0.259281
SVM	ElasticNet	8	0.166667	0.625	0.823413	0.833333	0.791398	0.862745	0.833333	0.25	1	0.453743
SVM	ElasticNet	9	0.111111	0.75	0.791667	0.888889	0.874074	0.902778	0.888889	0.5	1	0.661438
ttest	RandomForest	Validation	0.147059	0.852941	0.941176	0.852941	0.852814	0.854167	0.852941	0.882353	0.823529	0.707107
ttest	RandomForest	0	0.090909	0.823529	0.904575	0.909091	0.903813	0.909091	0.909091	0.666667	0.980392	0.727607
ttest	RandomForest	1	0.227273	0.664706	0.658824	0.772727	0.769912	0.767483	0.772727	0.466667	0.862745	0.337679
ttest	RandomForest	2	0.092593	0.880952	0.956349	0.907407	0.908701	0.910778	0.907407	0.833333	0.928571	0.740888
ttest	RandomForest	3	0.12963	0.827381	0.89881	0.87037	0.872182	0.874713	0.87037	0.75	0.904762	0.6367
ttest	RandomForest	4	0.185185	0.732143	0.819444	0.814815	0.814815	0.814815	0.814815	0.583333	0.880952	0.464286
ttest	RandomForest	5	0.203704	0.779762	0.771825	0.796296	0.807411	0.832362	0.796296	0.75	0.809524	0.500851
ttest	RandomForest	6	0.388889	0.422619	0.402778	0.611111	0.604945	0.599013	0.611111	0.083333	0.761905	− 0.15975
ttest	RandomForest	7	0.222222	0.64881	0.805556	0.777778	0.770261	0.765152	0.777778	0.416667	0.880952	0.318529
ttest	RandomForest	8	0.166667	0.77381	0.904762	0.833333	0.835663	0.838649	0.833333	0.666667	0.880952	0.532513
ttest	RandomForest	9	0.055556	0.904762	0.964286	0.944444	0.943564	0.943622	0.944444	0.833333	0.97619	0.835631
ttest	SVC	Validation	0.235294	0.764706	0.939446	0.764706	0.757143	0.802372	0.764706	0.941176	0.588235	0.565825
ttest	SVC	0	0.090909	0.847059	0.917647	0.909091	0.906718	0.906716	0.909091	0.733333	0.960784	0.731399
ttest	SVC	1	0.227273	0.664706	0.724183	0.772727	0.769912	0.767483	0.772727	0.466667	0.862745	0.337679
ttest	SVC	2	0.12963	0.857143	0.938492	0.87037	0.875171	0.88604	0.87037	0.833333	0.880952	0.662994
ttest	SVC	3	0.166667	0.833333	0.90873	0.833333	0.842427	0.866455	0.833333	0.833333	0.833333	0.596759
ttest	SVC	4	0.185185	0.761905	0.767857	0.814815	0.819679	0.826984	0.814815	0.666667	0.857143	0.496929
ttest	SVC	5	0.222222	0.767857	0.781746	0.777778	0.791453	0.824074	0.777778	0.75	0.785714	0.472456
ttest	SVC	6	0.444444	0.416667	0.382937	0.555556	0.57619	0.600877	0.555556	0.166667	0.666667	− 0.15174
ttest	SVC	7	0.203704	0.720238	0.740079	0.796296	0.799143	0.802585	0.796296	0.583333	0.857143	0.428326
ttest	SVC	8	0.203704	0.75	0.918651	0.796296	0.803841	0.816524	0.796296	0.666667	0.833333	0.464095
ttest	SVC	9	0.111111	0.869048	0.974206	0.888889	0.891807	0.897619	0.888889	0.833333	0.904762	0.700219
ttest	LogisticRegression	Validation	0.132353	0.867647	0.933391	0.867647	0.867389	0.870532	0.867647	0.823529	0.911765	0.738173
ttest	LogisticRegression	0	0.151515	0.666667	0.930719	0.848485	0.81737	0.873323	0.848485	0.333333	1	0.52791
ttest	LogisticRegression	1	0.19697	0.660784	0.720261	0.80303	0.787935	0.784903	0.80303	0.4	0.921569	0.375846
ttest	LogisticRegression	2	0.111111	0.809524	0.910714	0.888889	0.88513	0.884848	0.888889	0.666667	0.952381	0.662541
ttest	LogisticRegression	3	0.092593	0.791667	0.90873	0.907407	0.897825	0.917258	0.907407	0.583333	1	0.721995
ttest	LogisticRegression	4	0.222222	0.589286	0.759921	0.777778	0.748148	0.743056	0.777778	0.25	0.928571	0.236228
ttest	LogisticRegression	5	0.148148	0.785714	0.793651	0.851852	0.851852	0.851852	0.851852	0.666667	0.904762	0.571429
ttest	LogisticRegression	6	0.296296	0.482143	0.416667	0.703704	0.664198	0.636574	0.703704	0.083333	0.880952	− 0.04725
ttest	LogisticRegression	7	0.166667	0.654762	0.751984	0.833333	0.80543	0.828571	0.833333	0.333333	0.97619	0.443942
ttest	LogisticRegression	8	0.166667	0.714286	0.849206	0.833333	0.824302	0.822222	0.833333	0.5	0.928571	0.478091
ttest	LogisticRegression	9	0.111111	0.779762	0.980159	0.888889	0.880303	0.887681	0.888889	0.583333	0.97619	0.654802
ttest	Lasso	Validation	0.147059	0.852941	0.934256	0.852941	0.851787	0.864286	0.852941	0.764706	0.941176	0.717137
ttest	Lasso	0	0.166667	0.633333	0.918954	0.833333	0.7932	0.862903	0.833333	0.266667	1	0.468353
ttest	Lasso	1	0.181818	0.670588	0.717647	0.818182	0.800505	0.802233	0.818182	0.4	0.941176	0.416631
ttest	Lasso	2	0.111111	0.779762	0.918651	0.888889	0.880303	0.887681	0.888889	0.583333	0.97619	0.654802
ttest	Lasso	3	0.148148	0.666667	0.912698	0.851852	0.821256	0.875556	0.851852	0.333333	1	0.52915
ttest	Lasso	4	0.222222	0.589286	0.748016	0.777778	0.748148	0.743056	0.777778	0.25	0.928571	0.236228
ttest	Lasso	5	0.148148	0.755952	0.799603	0.851852	0.84684	0.844949	0.851852	0.583333	0.928571	0.547871
ttest	Lasso	6	0.259259	0.47619	0.406746	0.740741	0.661939	0.598291	0.740741	0	0.952381	− 0.10483
ttest	Lasso	7	0.148148	0.666667	0.746032	0.851852	0.821256	0.875556	0.851852	0.333333	1	0.52915
ttest	Lasso	8	0.185185	0.672619	0.859127	0.814815	0.800505	0.798309	0.814815	0.416667	0.928571	0.404027
ttest	Lasso	9	0.111111	0.75	0.980159	0.888889	0.874074	0.902778	0.888889	0.5	1	0.661438
ttest	ElasticNet	Validation	0.132353	0.867647	0.930796	0.867647	0.866928	0.875774	0.867647	0.794118	0.941176	0.743376
ttest	ElasticNet	0	0.166667	0.633333	0.922876	0.833333	0.7932	0.862903	0.833333	0.266667	1	0.468353
ttest	ElasticNet	1	0.19697	0.660784	0.717647	0.80303	0.787935	0.784903	0.80303	0.4	0.921569	0.375846
ttest	ElasticNet	2	0.111111	0.779762	0.914683	0.888889	0.880303	0.887681	0.888889	0.583333	0.97619	0.654802
ttest	ElasticNet	3	0.12963	0.708333	0.912698	0.87037	0.848668	0.888889	0.87037	0.416667	1	0.597614
ttest	ElasticNet	4	0.222222	0.589286	0.75	0.777778	0.748148	0.743056	0.777778	0.25	0.928571	0.236228
ttest	ElasticNet	5	0.148148	0.755952	0.799603	0.851852	0.84684	0.844949	0.851852	0.583333	0.928571	0.547871
ttest	ElasticNet	6	0.277778	0.464286	0.40873	0.722222	0.65233	0.594771	0.722222	0	0.928571	− 0.12964
ttest	ElasticNet	7	0.148148	0.666667	0.746032	0.851852	0.821256	0.875556	0.851852	0.333333	1	0.52915
ttest	ElasticNet	8	0.185185	0.672619	0.855159	0.814815	0.800505	0.798309	0.814815	0.416667	0.928571	0.404027
ttest	ElasticNet	9	0.111111	0.75	0.97619	0.888889	0.874074	0.902778	0.888889	0.5	1	0.661438

## Results

### Identification of dose and time-point to perform the feature selection

**Figure 1:**
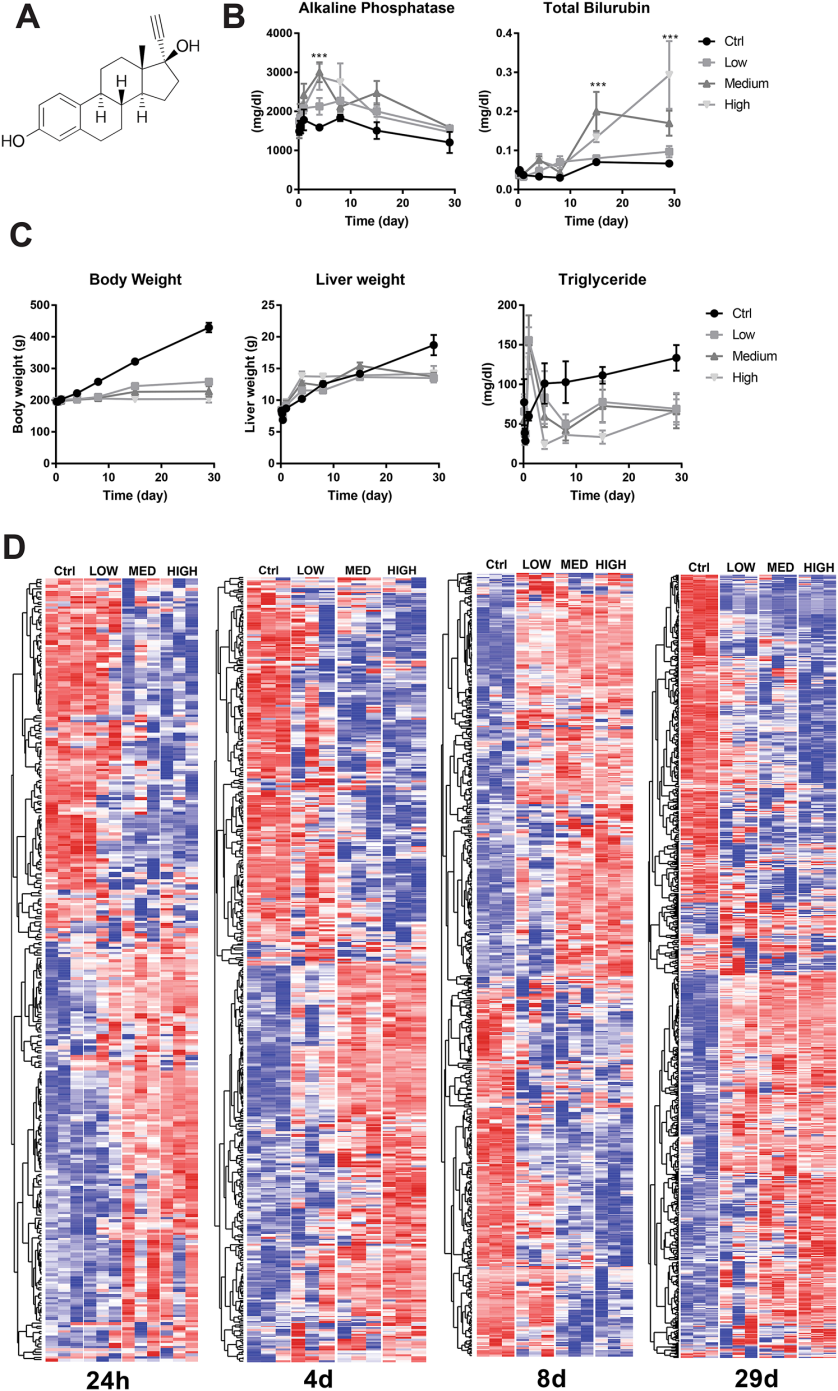
(**A**) Structure of ethinyl estradiol (EE). Image obtained from Wikipedia (https://commons.wikimedia.org/wiki/File:Ethinylestradiol.svg). (**B**) Serum alkaline phosphatase and total bilirubin levels of animals that are exposed to EE. Graphs are generated by Graphpad Prism8 software (GraphPad Software Inc., La Jolla, CA, www.graphpad.com). (**C**) Total body weight, liver weight and serum triglyceride levels of animals that are exposed to EE. Graphs are generated by Graphpad Prism8 software (GraphPad Software Inc., La Jolla, CA, www.graphpad.com). (**D**) Hierarchical clustering of hepatic genes regulated by low-, medium- and high-dose EE exposure at selected time points. Cluster3 software (https://bonsai.hgc.jp/~mdehoon/software/cluster/) was used for clustering the differentially expressed genes. Data was visualized using Treeview Java (https://jtreeview.sourceforge.net/).

To select the dose and time-point towards our goal of deriving a gene signature, we utilized the ethinyl estradiol (EE) dataset (Fig. 1A) as prolonged EE exposure causes hepatocellular carcinoma in rats. Glucuronide metabolite of EE is known to cause cholestatic hepatotoxicity by changing expression of ABCB11 and ABCC2 and disrupting bile flow and bile salt excretion^50^. In the TG-GATES data set, high-dose EE treatment caused a statistically significant change in clinical pathology parameters such as alkaline phosphatase by day 4, and total bilirubin levels by week 2 (Fig. 1B)^51^. Statistically significant body weight, liver weight and triglyceride changes were not detected until day 4 of the high dose EE treatment (Fig. 1C). Pathology analysis of hematoxylin and eosin (HE) images of liver samples showed that EE exposure resulted in hepatocyte necrosis, centrolobular hypertrophy, sinusoid dilatation, Kupffer cell proliferation and eosinophilic infiltration in periportal region. Necrosis was the only apical change that was common to livers that were exposed to any of the three different doses at earlier time point (4 days) (Table 4). We decided to focus on necrosis as an end-point, since it is predictive of liver carcinogenesis^52^. Next, we analyzed the dose response of gene expression across different time points (24 h, 4,8 and 29 days), which showed that manifestation of clinical pathologic indicators of liver damage, metabolic changes, and liver necrosis by high-dose EE exposure at the earlier time point was consistent with gene expression. Many genes were up- or downregulated in the liver by the high-dose EE exposure at all-time points assayed (Fig. 1D). Based on these observations, we focused on the high-dose exposure data to identify time points that will give us an early gene expression signature.

**Table 4:** Apical end-points related to ethinyl estradiol exposure.

Barcode	Exp_ID	Group_ID	Individual_ID	Compound_name	Dose_level	Sacrifice_period	Finding_type	Topography_type	
3.02E+09	305	10	3	Ethinylestradiol	Middle	8 day	Change, eosinophilic	Periportal	
3.02E+09	305	14	2	Ethinylestradiol	High	8 day	Change, eosinophilic	Periportal	
3.02E+09	305	14	4	Ethinylestradiol	High	8 day	Change, eosinophilic	Periportal	
No ChipData	305	14	1	Ethinylestradiol	High	8 day	Change, eosinophilic	Periportal	
No ChipData	305	14	5	Ethinylestradiol	High	8 day	Change, eosinophilic	Periportal	
3.02E+09	305	12	2	Ethinylestradiol	Middle	29 day	Change, eosinophilic	Periportal	
3.02E+09	305	12	3	Ethinylestradiol	Middle	29 day	Change, eosinophilic	Periportal	
3.02E+09	305	16	2	Ethinylestradiol	High	29 day	Change, eosinophilic	Periportal	
3.02E+09	305	16	4	Ethinylestradiol	High	29 day	Change, eosinophilic	Periportal	
3.02E+09	305	16	5	Ethinylestradiol	High	29 day	Change, eosinophilic	Periportal	
No ChipData	305	12	1	Ethinylestradiol	Middle	29 day	Change, eosinophilic	Periportal	
No ChipData	305	12	5	Ethinylestradiol	Middle	29 day	Change, eosinophilic	Periportal	
No ChipData	305	16	1	Ethinylestradiol	High	29 day	Change, eosinophilic	Periportal	
No ChipData	305	16	3	Ethinylestradiol	High	29 day	Change, eosinophilic	Periportal	
3.02E+09	305	11	5	Ethinylestradiol	Middle	15 day	Change, eosinophilic	Periportal	
3.02E+09	305	15	2	Ethinylestradiol	High	15 day	Change, eosinophilic	Periportal	
3.02E+09	305	15	3	Ethinylestradiol	High	15 day	Change, eosinophilic	Periportal	
3.02E+09	305	15	5	Ethinylestradiol	High	15 day	Change, eosinophilic	Periportal	
No ChipData	305	11	1	Ethinylestradiol	Middle	15 day	Change, eosinophilic	Periportal	
No ChipData	305	11	3	Ethinylestradiol	Middle	15 day	Change, eosinophilic	Periportal	
No ChipData	305	15	1	Ethinylestradiol	High	15 day	Change, eosinophilic	Periportal	
No ChipData	305	15	4	Ethinylestradiol	High	15 day	Change, eosinophilic	Periportal	
3.02E+09	305	16	2	Ethinylestradiol	High	29 day	Dilatation	Sinusoid	
No ChipData	305	16	1	Ethinylestradiol	High	29 day	Dilatation	Sinusoid	
No ChipData	305	16	3	Ethinylestradiol	High	29 day	Dilatation	Sinusoid	
3.02E+09	305	12	3	Ethinylestradiol	Middle	29 day	Hypertrophy	Centrilobular	
3.02E+09	305	16	2	Ethinylestradiol	High	29 day	Hypertrophy	Centrilobular	
3.02E+09	305	16	4	Ethinylestradiol	High	29 day	Hypertrophy	Centrilobular	
3.02E+09	305	16	5	Ethinylestradiol	High	29 day	Hypertrophy	Centrilobular	
No ChipData	305	12	5	Ethinylestradiol	Middle	29 day	Hypertrophy	Centrilobular	
No ChipData	305	16	1	Ethinylestradiol	High	29 day	Hypertrophy	Centrilobular	
No ChipData	305	16	3	Ethinylestradiol	High	29 day	Hypertrophy	Centrilobular	
3.02E+09	305	15	2	Ethinylestradiol	High	15 day	Hypertrophy	Centrilobular	
3.02E+09	305	15	5	Ethinylestradiol	High	15 day	Hypertrophy	Centrilobular	
3.02E+09	305	2	5	Ethinylestradiol	Control	8 day	Necrosis	Hepatocyte	
3.02E+09	305	10	3	Ethinylestradiol	Middle	8 day	Necrosis	Hepatocyte	
3.02E+09	305	14	3	Ethinylestradiol	High	8 day	Necrosis	Hepatocyte	
3.02E+09	305	9	2	Ethinylestradiol	Middle	4 day	Necrosis	Hepatocyte	
No ChipData	305	5	3	Ethinylestradiol	Low	4 day	Necrosis	Hepatocyte	
No ChipData	305	9	4	Ethinylestradiol	Middle	4 day	Necrosis	Hepatocyte	
No ChipData	305	13	5	Ethinylestradiol	High	4 day	Necrosis	Hepatocyte	
3.02E+09	305	8	4	Ethinylestradiol	Low	29 day	Necrosis	Hepatocyte	
No ChipData	305	8	3	Ethinylestradiol	Low	29 day	Necrosis	Hepatocyte	
3.02E+09	305	7	4	Ethinylestradiol	Low	15 day	Necrosis	Hepatocyte	
No ChipData	305	3	3	Ethinylestradiol	Control	15 day	Necrosis	Hepatocyte	
No ChipData	305	13	5	Ethinylestradiol	High	4 day	Proliferation, Kupffer cell		
3.02E+09	305	16	2	Ethinylestradiol	High	29 day	Proliferation, Kupffer cell		
3.02E+09	305	16	4	Ethinylestradiol	High	29 day	Proliferation, Kupffer cell		
No ChipData	305	16	3	Ethinylestradiol	High	29 day	Proliferation, Kupffer cell		
3.02E+09	305	15	3	Ethinylestradiol	High	15 day	Proliferation, Kupffer cell		
No ChipData	305	15	1	Ethinylestradiol	High	15 day	Proliferation, Kupffer cell		
No ChipData	305	13	5	Ethinylestradiol	High	4 day	Single cell necrosis	Hepatocyte	
No ChipData	305	16	3	Ethinylestradiol	High	29 day	Single cell necrosis	Hepatocyte	

**Figure 2:**
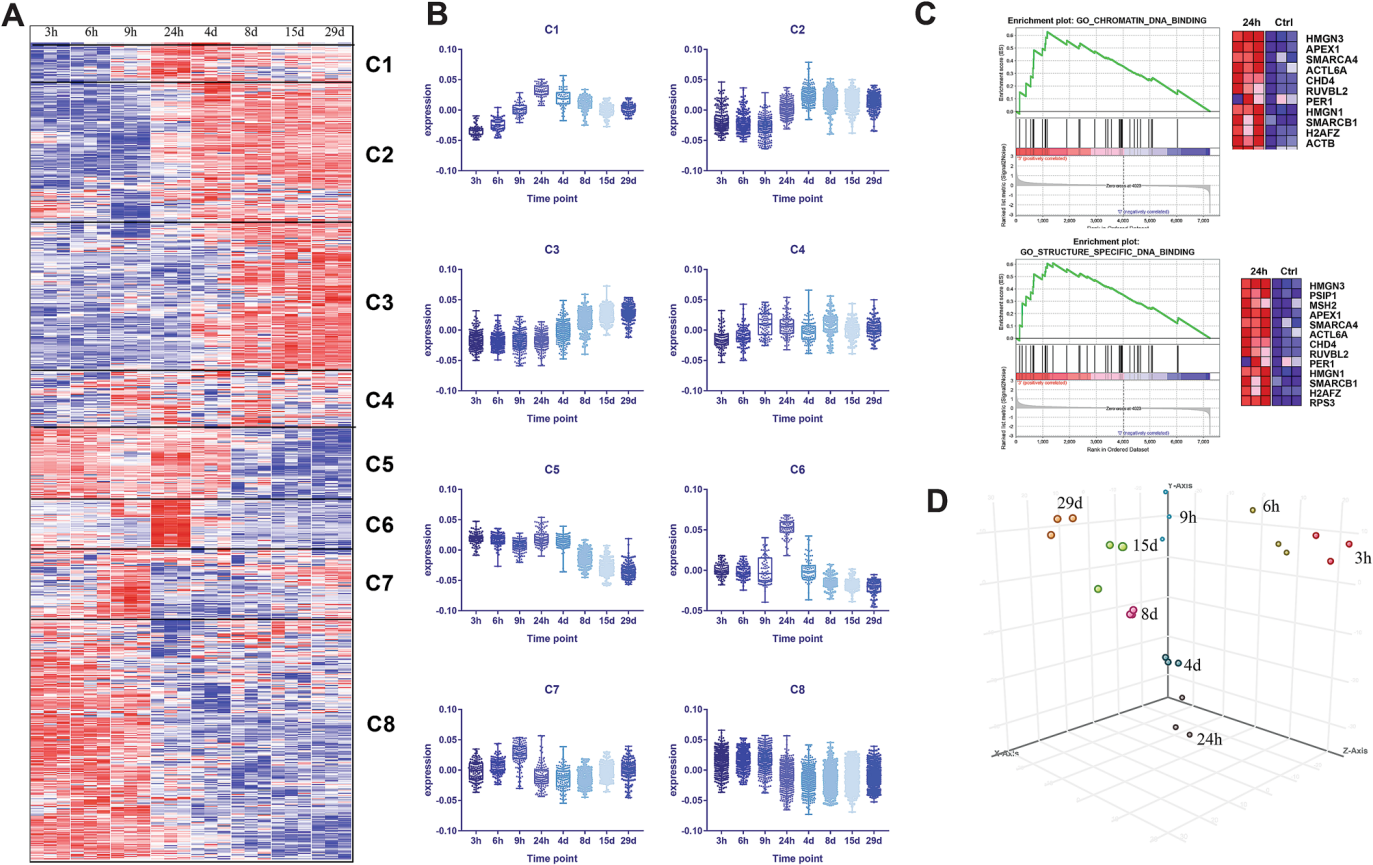
(**A**) Hierarchical clustering of hepatic genes that are regulated by high-dose EE exposure over 29 days. Cluster3 software (https://bonsai.hgc.jp/~mdehoon/software/cluster/) was used for clustering the differentially expressed genes. Data was visualized using Treeview Java (https://jtreeview.sourceforge.net/). (**B**) Gene expression patterns of clusters (C1–8) based on average gene expression values that were identified in 2A. Graphs are generated by Graphpad Prism8 software (GraphPad Software Inc., La Jolla, CA, www.graphpad.com). (**C**) GO terms that are significantly associated with C6. GSEA analysis was performed. Figures are generated using Gene Set Enrichment Analysis software (https://www.gsea-msigdb.org/gsea/index.jsp)^31,32^. (**D**) PCA analysis of hepatic gene regulation time course dataset for high-dose EE exposure. Figure was generated using StrandNGS (Version 3.1.1, Bangalore, India).

To identify the earliest time point data to be used in feature selection, we utilized 3, 6, 9, and 24 h and the 4, 8, 15 and 29 days’ time-points. Hierarchical clustering of 1387 differentially expressed genes identified eight clusters with distinct gene expression kinetics and function (C1–8, Fig. 2A–C and Supplementary Fig. 2). C1–4 were characterized by genes that were upregulated at later time points compared to earlier time points. C5 contained genes that were down-regulated at later time points by high-dose EE treatment. C6 had genes that were specifically upregulated at 24 h. These genes were involved in chromatin-DNA binding, potentially pointing out the primary transcriptional changes related to ethinyl estradiol exposure that would drive later liver toxicity. C7 and C8 contained genes that were upregulated at earlier times (3, 6 and 9 h of EE treatment). Principal component analysis of the data utilizing 1387 genes showed that different time points had a unique gene expression profile. Since 24 h time point was quite distinct from earlier time points in the PCA analysis and C6 indicated a robust gene expression program specific to this time point, we chose this time point for the further analysis (Fig. 2D). This time point was chosen for ensuing feature selection and classification since it has a distinct gene expression profile, and ensures expression and sufficient accumulation of markers.

### Gene expression feature reduction by differential expression analysis

**Figure 3:**
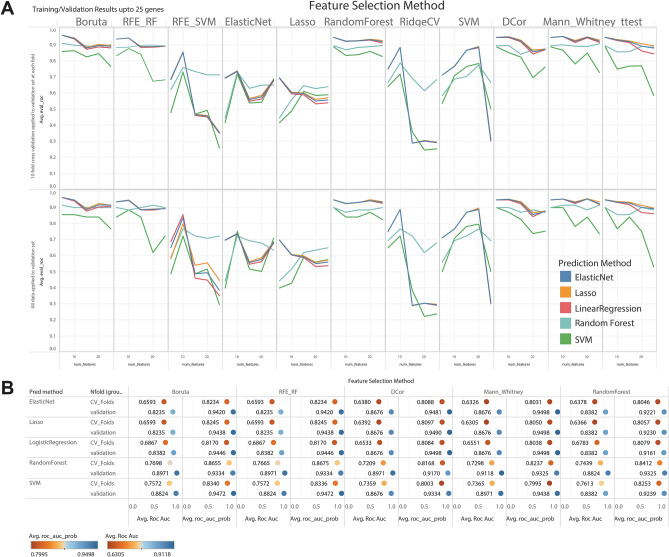
(**A**) Evaluation of average ROC for training (upper panel) and validation (lower panel) with increasing gene number for feature selection. (**B**) Comparison of ranges of average ROC values for different Nfold (groups) for each feature selection-prediction method combination. Both graphs are generated using Tableau software (Seattle, WA, USA, https://www.tableau.com/).

Our data (Figs. 1 and 2) generated using classical approaches to identify differentially expressed genes showed that we need to utilize more advanced statistical and computational approaches to reduce number of gene features that can discriminate between control and toxicant treated individuals, and to generate models that can predict with high accuracy if the toxicant exposure would result in future liver carcinogenesis. To achieve our goal and avoid overfitting or underfitting our data, we utilized the 24 h exposure microarray data for 42 compounds that result in necrosis from TGGATES database, and we performed feature selection from the 31,099 genes to identify a small set of features predictive of necrosis. We chose methods from filtering, wrapper and embedded approaches. Methods for feature selection included Mann–Whitney, t-test, DCor as filter methods; Boruta, RFE with both RF and SVM as wrapper methods; and RF, Elastic Net, Lasso, Ridge Regression Cross Validation (RidgeCV) and SVM as embedded methods (Table 2). When we tested AUC up to 50 (Supplementary Fig. 3A) or 100 (Supplementary Fig. 3B) features, accuracy in majority of models dropped off after 20 or 25 features (Fig. 3A). Thus, we chose the fewest features, top 10 genes that provided a level of desired high accuracy for each method.

**Figure 4:**
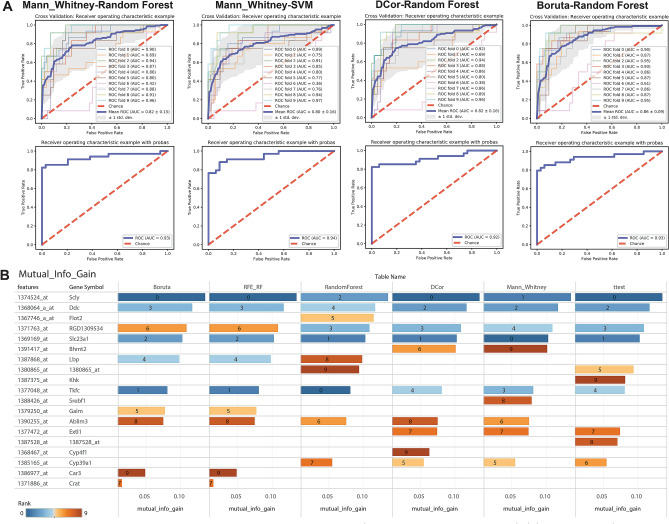
(**A**) ROC curves for training (upper) and validation (lower) datasets for best performing feature selection-prediction method combinations. (**B**) List of genes identified by six feature selection methods and their contribution to prediction methods as indicated by mutual info gain for each gene. Color shows details about Rank. The marks are labelled by rank. Both graphs are generated using Tableau software (Seattle, WA, USA, https://www.tableau.com/).

Given a set of 10 features from feature selection methods above, we conducted tenfold cross-validation (with all compounds grouped together in the same fold) utilizing the TG-GATEs dataset as training set, and MAQC-II dataset as an independent validation set. With this extensive testing and independent assessment, the gene signature that results is more likely to be a generalizable predictor. Based on ROC values, filter and wrapper feature selection methods in combination with Logistic Regression, RF and SVM performed with high accuracy (AUC > 0.75, F1 score > 0.75). To perform more detailed analysis, we focused on the four best performing feature selection methods (DCor, Boruta, RFE_RF, Mann–Whitney and Random Forest) and five classification methods (ElasticNet, Lasso, RF, SVM and Logistic Regression) (Fig. 3B) and unbiased performance error estimates of the models are obtained from the MAQC-II dataset (Table 5). The Mann–Whitney-RF combination had the highest F1 and MCC (F1 = 0.91, ROC = 0.91,sensitivity = 0.85, specificity = 0.97, MCC = 0.82), followed by Mann–Whitney-SVM (F1 = 0.89, ROC = 0.89,sensitivity = 0.88, specificity = 0.91, MCC = 0.79), Boruta and RF combination (F1 = 0.89, ROC = 0.89, sensitivity = 0.79, specificity = 0.1, MCC = 0.81), and DCor-RF (F1 = 0.89, ROC = 0,89,sensitivity = 0.82, specificity = 0.97, MCC = 0.80), (Fig. 4A, Tables 5 and 6). Overall, the top genes that contributed to the information were similar between Mann–Whitney, DCor and Boruta, five of the ten genes in the signature; Scly, Slc23a1, Dcd, Tkfc and RGD1309534, were the top contributors to the performance of the signature in all three methods used (Fig. 4B). Best performing feature selection method, Mann–Whitney, had Scly, Dcd, RGD1309534, Slc23a1, Bhmt2, Tkfc, Srebf1, Ablim3, Extl1 and Cyp39a1 genes (Fig. 4B).

**Table 5:** Comparison of the performance of various combinations of feature selection and classification methods.

Feature selection method	Prediction method	mse	roc_auc	roc_auc_prob	Accuracy	f1_score	Precision_score	Recall_score	Sensitivity	Specificity	mcc
Mann_Whitney	RandomForest	0.088235	0.911765	0.932526	0.911765	0.911458	0.917544	0.911765	0.852941	0.970588	0.829288
Mann_Whitney	SVM	0.102941	0.897059	0.943772	0.897059	0.897037	0.897403	0.897059	0.882353	0.911765	0.794461
Boruta	RandomForest	0.102941	0.897059	0.933391	0.897059	0.895956	0.914634	0.897059	0.794118	1	0.811503
DCor	RandomForest	0.102941	0.897059	0.916955	0.897059	0.896499	0.905836	0.897059	0.823529	0.970588	0.802846
Boruta	SVM	0.117647	0.882353	0.947232	0.882353	0.882251	0.883681	0.882353	0.852941	0.911765	0.766032
RFE_RF	SVM	0.117647	0.882353	0.947232	0.882353	0.882251	0.883681	0.882353	0.852941	0.911765	0.766032
RandomForest	RandomForest	0.117647	0.882353	0.932526	0.882353	0.88143	0.894643	0.882353	0.794118	0.970588	0.776899
DCor	LogisticRegression	0.132353	0.867647	0.949827	0.867647	0.865287	0.895349	0.867647	0.735294	1	0.762493
Mann_Whitney	LogisticRegression	0.132353	0.867647	0.949827	0.867647	0.865287	0.895349	0.867647	0.735294	1	0.762493
Mann_Whitney	Lasso	0.132353	0.867647	0.949827	0.867647	0.865287	0.895349	0.867647	0.735294	1	0.762493
Mann_Whitney	ElasticNet	0.132353	0.867647	0.949827	0.867647	0.865287	0.895349	0.867647	0.735294	1	0.762493
DCor	Lasso	0.132353	0.867647	0.948962	0.867647	0.865287	0.895349	0.867647	0.735294	1	0.762493
DCor	ElasticNet	0.132353	0.867647	0.948097	0.867647	0.865287	0.895349	0.867647	0.735294	1	0.762493
ttest	LogisticRegression	0.132353	0.867647	0.933391	0.867647	0.867389	0.870532	0.867647	0.823529	0.911765	0.738173
DCor	SVM	0.132353	0.867647	0.933391	0.867647	0.867618	0.867965	0.867647	0.882353	0.852941	0.735612
ttest	ElasticNet	0.132353	0.867647	0.930796	0.867647	0.866928	0.875774	0.867647	0.794118	0.941176	0.743376
ttest	RandomForest	0.147059	0.852941	0.941176	0.852941	0.852814	0.854167	0.852941	0.882353	0.823529	0.707107
ttest	Lasso	0.147059	0.852941	0.934256	0.852941	0.851787	0.864286	0.852941	0.764706	0.941176	0.717137
Boruta	LogisticRegression	0.161765	0.838235	0.944637	0.838235	0.835351	0.863721	0.838235	0.705882	0.970588	0.701493
RFE_RF	LogisticRegression	0.161765	0.838235	0.944637	0.838235	0.835351	0.863721	0.838235	0.705882	0.970588	0.701493
RandomForest	SVM	0.161765	0.838235	0.923875	0.838235	0.836503	0.853207	0.838235	0.735294	0.941176	0.69128
RandomForest	Lasso	0.161765	0.838235	0.92301	0.838235	0.833889	0.877778	0.838235	0.676471	1	0.71492
RandomForest	ElasticNet	0.161765	0.838235	0.922145	0.838235	0.833889	0.877778	0.838235	0.676471	1	0.71492
RandomForest	LogisticRegression	0.161765	0.838235	0.91609	0.838235	0.833889	0.877778	0.838235	0.676471	1	0.71492
Boruta	Lasso	0.176471	0.823529	0.943772	0.823529	0.819629	0.854167	0.823529	0.676471	0.970588	0.677003
RFE_RF	Lasso	0.176471	0.823529	0.943772	0.823529	0.819629	0.854167	0.823529	0.676471	0.970588	0.677003
Boruta	ElasticNet	0.176471	0.823529	0.942042	0.823529	0.819629	0.854167	0.823529	0.676471	0.970588	0.677003
RFE_RF	ElasticNet	0.176471	0.823529	0.942042	0.823529	0.819629	0.854167	0.823529	0.676471	0.970588	0.677003
ElasticNet_alpha_.001	LogisticRegression	0.220588	0.779412	0.741349	0.779412	0.771044	0.827254	0.779412	0.588235	0.970588	0.604777
ttest	SVM	0.235294	0.764706	0.939446	0.764706	0.757143	0.802372	0.764706	0.941176	0.588235	0.565825
RidgeCV	RandomForest	0.235294	0.764706	0.82699	0.764706	0.763889	0.768421	0.764706	0.705882	0.823529	0.533114
RFE_SVM	RandomForest	0.235294	0.764706	0.817474	0.764706	0.761404	0.78022	0.764706	0.647059	0.882353	0.544705
ElasticNet_alpha_.001	Lasso	0.235294	0.764706	0.736159	0.764706	0.759505	0.789773	0.764706	0.617647	0.911765	0.553912
ElasticNet_alpha_.001	ElasticNet	0.235294	0.764706	0.734429	0.764706	0.759505	0.789773	0.764706	0.617647	0.911765	0.553912
ElasticNet_alpha_.001	SVM	0.25	0.75	0.762111	0.75	0.748641	0.755526	0.75	0.676471	0.823529	0.505496
SVM	LogisticRegression	0.264706	0.735294	0.760381	0.735294	0.71978	0.802222	0.735294	0.5	0.970588	0.533333
RidgeCV	SVM	0.279412	0.720588	0.874567	0.720588	0.709989	0.758359	0.720588	0.529412	0.911765	0.477455
RFE_SVM	SVM	0.279412	0.720588	0.865052	0.720588	0.696927	0.820755	0.720588	0.441176	1	0.531995
ElasticNet_alpha_.001	RandomForest	0.279412	0.720588	0.75519	0.720588	0.719069	0.725464	0.720588	0.794118	0.647059	0.446026
SVM	SVM	0.294118	0.705882	0.776817	0.705882	0.704861	0.708772	0.705882	0.647059	0.764706	0.414644
SVM	RandomForest	0.308824	0.691176	0.831315	0.691176	0.658618	0.809091	0.691176	0.382353	1	0.486172
RFE_SVM	ElasticNet	0.323529	0.676471	0.851211	0.676471	0.638647	0.803571	0.676471	0.352941	1	0.46291
RFE_SVM	Lasso	0.323529	0.676471	0.849481	0.676471	0.638647	0.803571	0.676471	0.352941	1	0.46291
RFE_SVM	LogisticRegression	0.323529	0.676471	0.845156	0.676471	0.638647	0.803571	0.676471	0.352941	1	0.46291
SVM	Lasso	0.323529	0.676471	0.763841	0.676471	0.645833	0.769841	0.676471	0.382353	0.970588	0.436436
SVM	ElasticNet	0.323529	0.676471	0.763841	0.676471	0.645833	0.769841	0.676471	0.382353	0.970588	0.436436
RidgeCV	Lasso	0.367647	0.632353	0.885813	0.632353	0.58486	0.744019	0.632353	0.294118	0.970588	0.359425
Lasso_alpha_.001	LogisticRegression	0.367647	0.632353	0.627163	0.632353	0.631636	0.633391	0.632353	0.588235	0.676471	0.265742
RidgeCV	ElasticNet	0.382353	0.617647	0.884948	0.617647	0.563241	0.734483	0.617647	0.264706	0.970588	0.332182
RidgeCV	LogisticRegression	0.397059	0.602941	0.865917	0.602941	0.540885	0.724105	0.602941	0.235294	0.970588	0.303774
ElasticNet_alpha_.01	RandomForest	0.397059	0.602941	0.676471	0.602941	0.587879	0.620567	0.602941	0.794118	0.411765	0.222812
Lasso_alpha_.001	Lasso	0.426471	0.573529	0.605536	0.573529	0.573437	0.573593	0.573529	0.588235	0.558824	0.147122
Lasso_alpha_.001	ElasticNet	0.426471	0.573529	0.602941	0.573529	0.573437	0.573593	0.573529	0.588235	0.558824	0.147122
Lasso_alpha_.001	RandomForest	0.470588	0.529412	0.663495	0.529412	0.484848	0.544974	0.529412	0.823529	0.235294	0.072739
ElasticNet_alpha_.01	SVM	0.5	0.5	0.545848	0.5	0.333333	0.25	0.5	1	0	0
Lasso_alpha_.01	RandomForest	0.514706	0.485294	0.474048	0.485294	0.326733	0.246269	0.485294	0.970588	0	− 0.12217
ElasticNet_alpha_.01	Lasso	0.558824	0.441176	0.562284	0.441176	0.416441	0.429167	0.441176	0.647059	0.235294	− 0.1291
ElasticNet_alpha_.01	ElasticNet	0.558824	0.441176	0.557958	0.441176	0.416441	0.429167	0.441176	0.647059	0.235294	− 0.1291
Lasso_alpha_.001	SVM	0.573529	0.426471	0.605536	0.426471	0.366005	0.381119	0.426471	0.735294	0.117647	− 0.18699
ElasticNet_alpha_.01	LogisticRegression	0.588235	0.411765	0.553633	0.411765	0.328063	0.324138	0.411765	0.764706	0.058824	− 0.24914
Lasso_alpha_.01	SVM	0.838235	0.161765	0.110727	0.161765	0.139241	0.122222	0.161765	0.323529	0	− 0.71492
Lasso_alpha_.01	LogisticRegression	0.867647	0.132353	0.122837	0.132353	0.116883	0.104651	0.132353	0.264706	0	− 0.76249
Lasso_alpha_.01	Lasso	0.867647	0.132353	0.119377	0.132353	0.116883	0.104651	0.132353	0.264706	0	− 0.76249
Lasso_alpha_.01	ElasticNet	0.867647	0.132353	0.117647	0.132353	0.116883	0.104651	0.132353	0.264706	0	− 0.76249

For methods that produced a regressive score, such as Lasso and ElasticNet, we chose 0.5 as the split point to make a binary classification prediction.

**Table 6:** Performance of best four combinations of feature selection and classification methods.

Feature selection method	Prediction method	nfold	mse	roc_auc	roc_auc_prob	Accuracy	f1_score	Precision_score	Recall_score	Sensitivity	Specificity	mcc
Mann_Whitney	RandomForest	Validation	0.088235	0.911765	0.932526	0.911765	0.911458	0.917544	0.911765	0.852941	0.970588	0.829288
Mann_Whitney	RandomForest	9	0.092593	0.85119	0.962302	0.907407	0.905939	0.905332	0.907407	0.75	0.952381	0.725032
Mann_Whitney	RandomForest	8	0.092593	0.880952	0.906746	0.907407	0.908701	0.910778	0.907407	0.833333	0.928571	0.740888
Mann_Whitney	RandomForest	7	0.203704	0.660714	0.878968	0.796296	0.785258	0.780247	0.796296	0.416667	0.904762	0.358569
Mann_Whitney	RandomForest	6	0.425926	0.39881	0.420635	0.574074	0.580027	0.5862	0.574074	0.083333	0.714286	− 0.1968
Mann_Whitney	RandomForest	5	0.185185	0.791667	0.801587	0.814815	0.823413	0.841374	0.814815	0.75	0.833333	0.531105
Mann_Whitney	RandomForest	4	0.203704	0.720238	0.861111	0.796296	0.799143	0.802585	0.796296	0.583333	0.857143	0.428326
Mann_Whitney	RandomForest	3	0.203704	0.720238	0.869048	0.796296	0.799143	0.802585	0.796296	0.583333	0.857143	0.428326
Mann_Whitney	RandomForest	2	0.148148	0.785714	0.944444	0.851852	0.851852	0.851852	0.851852	0.666667	0.904762	0.571429
Mann_Whitney	RandomForest	1	0.212121	0.67451	0.69281	0.787879	0.782343	0.778467	0.787879	0.466667	0.882353	0.367765
Mann_Whitney	RandomForest	0	0.106061	0.813725	0.899346	0.893939	0.889562	0.890572	0.893939	0.666667	0.960784	0.681747
Mann_Whitney	SVM	Validation	0.102941	0.897059	0.943772	0.897059	0.897037	0.897403	0.897059	0.882353	0.911765	0.794461
Mann_Whitney	SVM	9	0.111111	0.839286	0.968254	0.888889	0.888889	0.888889	0.888889	0.75	0.928571	0.678571
Mann_Whitney	SVM	8	0.092593	0.910714	0.94246	0.907407	0.910837	0.920798	0.907407	0.916667	0.904762	0.762443
Mann_Whitney	SVM	7	0.203704	0.660714	0.763889	0.796296	0.785258	0.780247	0.796296	0.416667	0.904762	0.358569
Mann_Whitney	SVM	6	0.481481	0.363095	0.363095	0.518519	0.540873	0.56652	0.518519	0.083333	0.642857	− 0.24929
Mann_Whitney	SVM	5	0.166667	0.803571	0.765873	0.833333	0.839506	0.851282	0.833333	0.75	0.857143	0.563545
Mann_Whitney	SVM	4	0.222222	0.708333	0.795635	0.777778	0.783615	0.791667	0.777778	0.583333	0.833333	0.395285
Mann_Whitney	SVM	3	0.203704	0.779762	0.847222	0.796296	0.807411	0.832362	0.796296	0.75	0.809524	0.500851
Mann_Whitney	SVM	2	0.166667	0.803571	0.911706	0.833333	0.839506	0.851282	0.833333	0.75	0.857143	0.563545
Mann_Whitney	SVM	1	0.227273	0.688235	0.74902	0.772727	0.775268	0.778182	0.772727	0.533333	0.843137	0.368143
Mann_Whitney	SVM	0	0.151515	0.807843	0.887582	0.848485	0.851705	0.856706	0.848485	0.733333	0.882353	0.590021
DCor	RandomForest	Validation	0.102941	0.897059	0.916955	0.897059	0.896499	0.905836	0.897059	0.823529	0.970588	0.802846
DCor	RandomForest	9	0.111111	0.809524	0.958333	0.888889	0.88513	0.884848	0.888889	0.666667	0.952381	0.662541
DCor	RandomForest	8	0.092593	0.880952	0.886905	0.907407	0.908701	0.910778	0.907407	0.833333	0.928571	0.740888
DCor	RandomForest	7	0.203704	0.660714	0.857143	0.796296	0.785258	0.780247	0.796296	0.416667	0.904762	0.358569
DCor	RandomForest	6	0.425926	0.39881	0.380952	0.574074	0.580027	0.5862	0.574074	0.083333	0.714286	− 0.1968
DCor	RandomForest	5	0.185185	0.791667	0.791667	0.814815	0.823413	0.841374	0.814815	0.75	0.833333	0.531105
DCor	RandomForest	4	0.203704	0.720238	0.865079	0.796296	0.799143	0.802585	0.796296	0.583333	0.857143	0.428326
DCor	RandomForest	3	0.203704	0.720238	0.875	0.796296	0.799143	0.802585	0.796296	0.583333	0.857143	0.428326
DCor	RandomForest	2	0.148148	0.785714	0.938492	0.851852	0.851852	0.851852	0.851852	0.666667	0.904762	0.571429
DCor	RandomForest	1	0.212121	0.65098	0.696732	0.787879	0.775564	0.770248	0.787879	0.4	0.901961	0.33955
DCor	RandomForest	0	0.106061	0.790196	0.917647	0.893939	0.885811	0.894481	0.893939	0.6	0.980392	0.678357
Boruta	RandomForest	Validation	0.102941	0.897059	0.933391	0.897059	0.895956	0.914634	0.897059	0.794118	1	0.811503
Boruta	RandomForest	9	0.092593	0.880952	0.954365	0.907407	0.908701	0.910778	0.907407	0.833333	0.928571	0.740888
Boruta	RandomForest	8	0.111111	0.839286	0.865079	0.888889	0.888889	0.888889	0.888889	0.75	0.928571	0.678571
Boruta	RandomForest	7	0.203704	0.660714	0.855159	0.796296	0.785258	0.780247	0.796296	0.416667	0.904762	0.358569
Boruta	RandomForest	6	0.407407	0.410714	0.605159	0.592593	0.592593	0.592593	0.592593	0.083333	0.738095	− 0.17857
Boruta	RandomForest	5	0.185185	0.791667	0.865079	0.814815	0.823413	0.841374	0.814815	0.75	0.833333	0.531105
Boruta	RandomForest	4	0.148148	0.815476	0.863095	0.851852	0.855743	0.862302	0.851852	0.75	0.880952	0.598574
Boruta	RandomForest	3	0.12963	0.857143	0.934524	0.87037	0.875171	0.88604	0.87037	0.833333	0.880952	0.662994
Boruta	RandomForest	2	0.092593	0.910714	0.950397	0.907407	0.910837	0.920798	0.907407	0.916667	0.904762	0.762443
Boruta	RandomForest	1	0.19697	0.707843	0.866667	0.80303	0.80059	0.798576	0.80303	0.533333	0.882353	0.426119
Boruta	RandomForest	0	0.090909	0.823529	0.895425	0.909091	0.903813	0.909091	0.909091	0.666667	0.980392	0.727607

## Discussion

In this study, we built a ML-based predictive process composed of ten genes that should be regulated in rat liver after 24 h of toxicant exposure and accurately predicts a liver necrosis phenotype, an indicator of liver carcinogenicity after long-term molecule exposure^52^. We compared various feature selection and classification methods to identify early gene biomarkers of liver toxicity using an extensive gene expression database, TG-GATEs and an independent validation dataset, MAQC II. Initially, we focused on necrosis, which is a valid end point to predict liver cancer^52^ as necrotic cell death is a common feature in liver disease^53–55^. Given that necrosis is a fairly common end point for adverse processes, we anticipate that our methods are applicable to other apical end-points. Rather than depending solely on the parametric models, the methods utilized in the feature selection and predictive analysis are adaptive, and involve models requiring the optimization of a tuning or smoothing parameter to control the trade-off between model generality and complexity. Appropriate choice of tuning parameters is critical for feature selection stability and good performance of the resulting predictive model estimator. TG-GATEs microarray gene expression data contains few samples (n) and very large features or genes (p). In machine learning, this p ≫ n problem usually has major consequences for prediction modeling. For example, over fitting may occur, which can cause unreliability for the prediction model to be used on other data sets ^56^. Our study design with an extensive, independent validation and careful feature selection and curation, likely overcomes this hurdle.

Parameter tuning has traditionally been a manual task because of the limited number of trials. Recently, it has been shown automated pre-tuning surrogate-based parameter optimization was successfully applied in the learning for a wide variety of feature selector/classifiers^57,58^ and to deep belief networks^59,60^. These methods combined computational power with model building about the behavior of the error function in the parameter space, and they improve on manual parameter tuning. To improve the performance of our feature selection and predictive analysis steps we utilized MAQCII-NIEHS (GSE16716) dataset as the surrogate for pre tuning the parameters of these methods^17^. Since we used an independent validation set (MAQCII) to select prediction models with higher accuracy, we avoided overfitting issues that typically afflict studies that only employ cross-validation. We also utilized methods that dealt directly with binary classification rather than regressive methods to generally predict multiple apical end-points from the TG-GATEs database.

We have previously used t-test and RF coupled with logistic regression to identify biomarkers of breast cancer risk^61^. The dataset we used contained much less features from a smaller population. Since, in our study we are dealing with many more features from larger number of experiment we used an expanded list of feature selection methods that fall into one of the three main categories: Mann–Whitney, t-test, DCor as filter methods; Boruta, RFE with both RF and SVM as wrapper methods; and RF, Elastic Net, Lasso, Ridge Regression Cross Validation (RidgeCV) and SVM as embedded methods. For assessing classification performance we used logistic regression, RF, and support vector machine (SVM), Lasso and ElasticNet. Instead of relying on one machine learning method^7–9^, we used an exhaustive approach wherein we have compared combinations of aforementioned feature selection and classification methods and tested their performance rigorously on a validation set. Our process addresses several limitations of traditional methods for *multimodal* signature studies in terms of data handling (the number of features are orders of magnitude greater than the number of samples, there are heterogeneous features from different modalities, and there are multiple phenotypic responses to the same conditions) as well as procedural (increased performance over a single approach and assessment of key features in the context of phenotype)^35,36^. The net outcomes were that we obtained a minimal descriptive set of 10 biomarkers (key star features) related to liver toxicity (specifically, necrosis), a ranked list of biomarkers that describe a phenotype, a classifier useful for toxicity screening, a confidence measure for the classifier, and a classifier performance evaluated on MACQII data unseen during training^43,62,63^. Number of features used for classification is very low, which avoids the problem of overfitting. In addition, we used an iterative process where we selected features and tested their performance on the validation set. This exhaustive process ensured that only best predictors with minimum number of genes were used and that their performance was validated in an independent dataset (MAQCII) to avoid low reproducibility of identified biomarkers.

To avoid overfitting while building our prediction models and to eventually utilize the biomarker genes in a practical laboratory test for unknown chemicals, we limited our gene list to 10 candidates. The genes that were selected with various methods are involved in metabolism and detoxification (Car3, Crat, Cyp39a1, Dcd, Lbp, Scly, Slc23a1, and Tkfc) and transcriptional regulation (Ablim3, Srebf1). Several of these genes were implicated in liver carcinogenesis including Crat^64^, Car3^65^ and Slc23a1^66^.

In summary, using feature selection, modeling and validation with an independent data set, we found a robust set of genes that appeared to be broadly generalizable for prediction. We selected the top genes and the best models to predict whether a compound would cause liver necrosis. This selected pipeline provided predictions with high accuracy. Given the broad set of conditions and a manageable set of predictor genes, we anticipate that this signature can be used to predict future carcinogenic effects of long-term exposure to liver toxicants in rodent models and accelerate the predictability of toxic effects in humans.

## Supplementary information

Supplementary Information.

## Data Availability

The datasets analyzed during the current study are available in the Life Science Database Archive, https://dbarchive.biosciencedbc.jp/en/open-tggates/download.html. A public GitHub repository with datasets and code is available here: https://github.com/brandis2/TG-GATES.

## Abbreviations

MAQC—II: Microarray quality control—II
TG-GATES: Toxicogenomics project-genomics assisted toxicity evaluation system
ML: Machine learning
RFE: Recursive feature elimination
SVM: Support vector machine
RF: Random forest
RidgeCV: Ridge regression cross validation
ROC: Receiver operating characteristic
RMA: Robust multi-array
