# Supplementary material for: Identification of early liver toxicity gene biomarkers using comparative supervised machine learning
Source: Sci Rep. 2020 Nov 5;10:19128. doi: 10.1038/s41598-020-76129-8 (PMC7645727; doi:10.1038/s41598-020-76129-8)

Supplementary Materials and Legends

Identification of Early Liver Toxicity Gene Biomarkers Using Comparative Supervised Machine Learning

Brandi Patrice Smith[1,2](brandis2@illinois.edu), Loretta Sue Auvil[3](lauvil@illinois.edu), Michael Welge[3,4](mwelge@illinois.edu), Colleen Bushell[3,4,5](cbushell@illinois.edu) ,Rohit Bhargava[6,8,9](rxb@illinois.edu), Navin Elango[7](navin.elango@corteva.com), Kamin Johnson[7](kamin.johnson@corteva.com), Zeynep Madak-Erdogan*[1,3,4,8,9](zmadake2@illinois.edu)

[1] Department of Food Science and Human Nutrition, University of Illinois, Urbana-Champaign, Urbana, IL, USA

[2] Illinois Informatics Institute, University of Illinois, Urbana-Champaign, Urbana, IL USA

[3] National Center for Supercomputing Applications, University of Illinois, Urbana-Champaign, Urbana, IL, USA

[4] Carl R. Woese Institute for Genomic Biology, University of Illinois, Urbana-Champaign, Urbana, IL, USA

[5] Carle Illinois College of Medicine, University of Illinois, Urbana-Champaign, Urbana, IL, USA

[6]Department of Bioengineering, University of Illinois, Urbana-Champaign, Urbana, IL, USA

[7] Corteva Agrisciences, The Agriculture Division of DowDupont,Indianapolis, IN, USA

[8] Cancer Center at Illinois, University of Illinois, Urbana-Champaign, Urbana, IL,USA

[9] Beckman Institute for Advanced Science and Technology, University of Illinois at Urbana-Champaign, Urbana, IL, USA

Supplementary Figure 1: A. Overview of the compounds in TG-GATES data. **B.** Experimental design. Timeline and doses used for experiments in TG-GATES database **C.** Flow chart for development of the ML pipeline.

All graphs are generated using Graph is generated using Powerpoint software (Microsoft Corporation, Seattle, WA, USA).

Supplementary Figure 2: GSEA analysis of clusters related to Figure 2. Upper panel graphs are generated by Graphpad© Prism8 software (GraphPad Software Inc., La Jolla, CA, www.graphpad.com). Lower panels are generated using Gene Set Enrichment Analysis software (https://www.gsea-msigdb.org/gsea/index.jsp) [31,32].

Supplementary Figure 3: Evaluation of average ROC for gene sets up to **A.** 50 and **B.** 100 . Figures are generated using Tableau software (Seattle, WA, USA, https://www.tableau.com/).

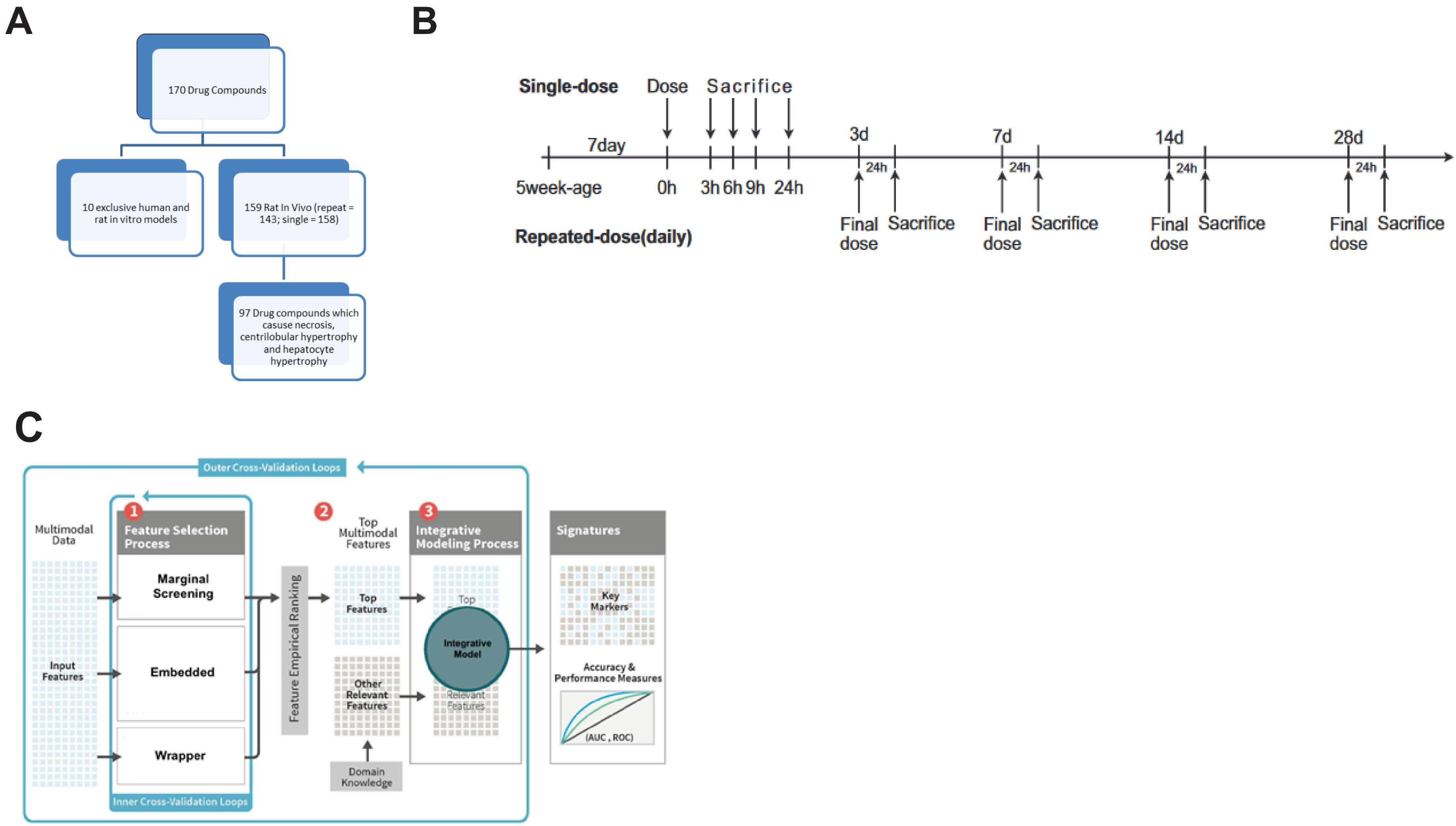

A
170 Drug Compounds
10 exclusive human and rat in vitro models
159 Rat In Vivo (repeat = 143; single = 158)
97 Drug compounds which casuse necrosis, centrilobular hypertrophy and hepatocyte hypertrophy
B
Single-dose
Dose
Sacrifice
7day
5week-age
0h
3h
6h
9h
24h
3d
7d
14d
28d
24h
Final dose
Sacrifice
Repeated-dose(daily)
C
Outer Cross-Validation Loops
Multimodal Data
Input Features
1
Feature Selection Process
Marginal Screening
Embedded
Wrapper
Inner Cross-Validation Loops
Feature Empirical Ranking
2
Top Multimodal Features
Top Features
Other Relevant Features
Domain Knowledge
3
Integrative Modeling Process
Integrative Model
Signatures
Key Markers
Accuracy & Performance Measures
(AUC , ROC)

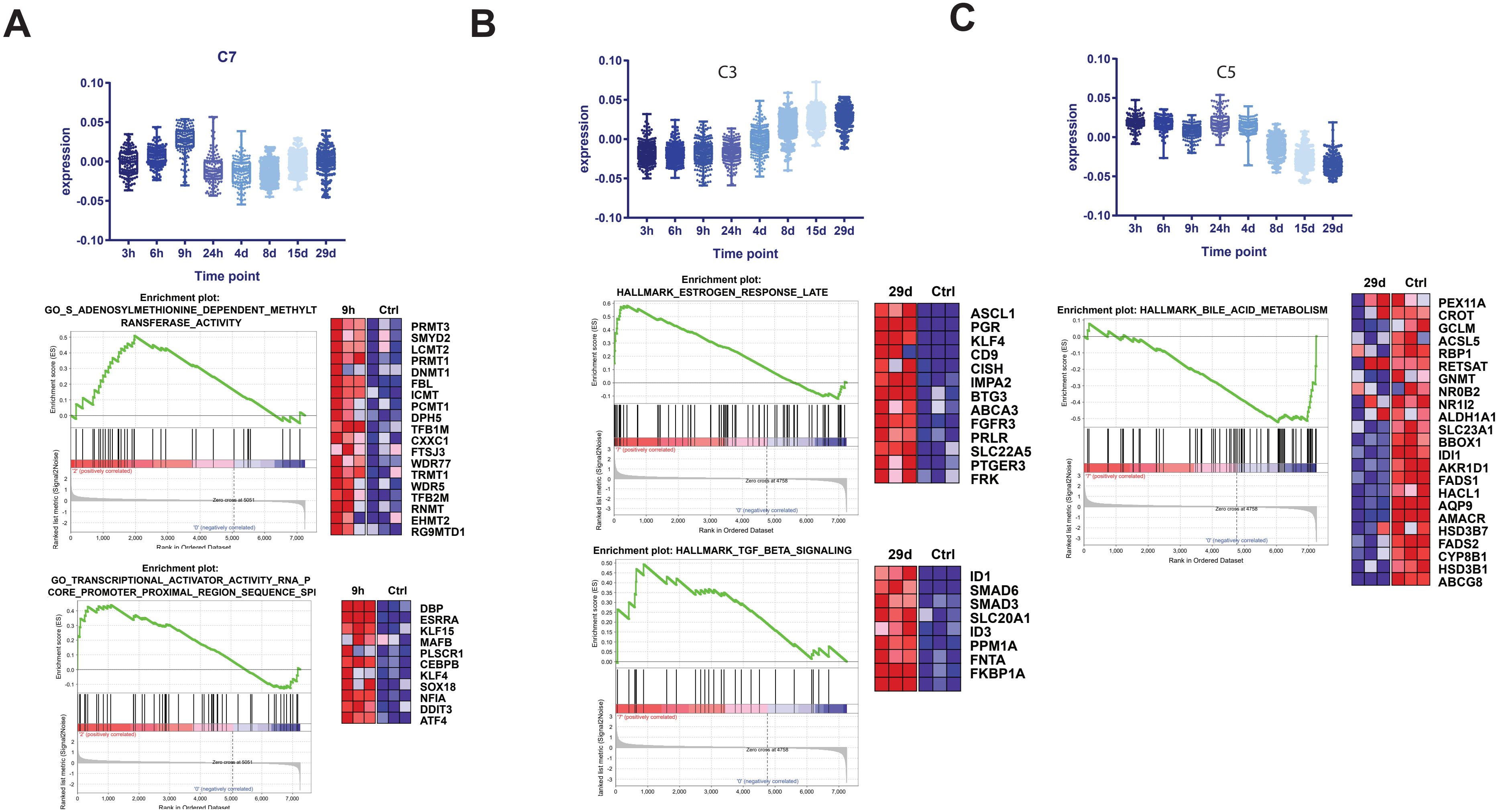
A
B
C
C7
C3
C5
expression
Time point
3h
6h
9h
24h
4d
8d
15d
29d
Enrichment plot:
GO_S_ADENOSYLMETHIONINE_DEPENDENT_METHYLTRANSFERASE_ACTIVITY
9h
Ctrl
PRMT3
SMYD2
LCMT2
PRMT1
DNMT1
FBL
ICMT
PCMT1
DPH5
TFB1M
CXXC1
FTSJ3
WDR77
TRMT1
WDR5
TFB2M
RNMT
EHMT2
RG9MTD1
Enrichment plot:
GO_TRANSCRIPTIONAL_ACTIVATOR_ACTIVITY_RNA_POLYMERASE_II_CORE_PROMOTER_PROXIMAL_REGION_SEQUENCE_SPI
9h
Ctrl
DBP
ESRRA
KLF15
MAFB
PLSCR1
CEBPB
KLF4
SOX18
NFIA
DDIT3
ATF4
Enrichment plot:
HALLMARK_ESTROGEN_RESPONSE_LATE
29d
Ctrl
ASCL1
PGR
KLF4
CD9
CISH
IMPA2
BTG3
ABCA3
FGFR3
PRLR
SLC22A5
PTGER3
FRK
Enrichment plot: HALLMARK_TGF_BETA_SIGNALING
29d
Ctrl
ID1
SMAD6
SMAD3
SLC20A1
ID3
PPM1A
FNTA
FKBP1A
Enrichment plot: HALLMARK_BILE_ACID_METABOLISM
29d
Ctrl
PEX11A
CROT
GCLM
ACSL5
RBP1
RETSAT
GNMT
NR0B2
NR1I2
ALDH1A1
SLC23A1
BBOX1
IDI1
AKR1D1
FADS1
HACL1
AQP9
AMACR
HSD3B7
FADS2
CYP8B1
HSD3B1
ABCG8
Enrichment score (ES)
Ranked list metric (Signal2Noise)
Rank in Ordered Dataset

A

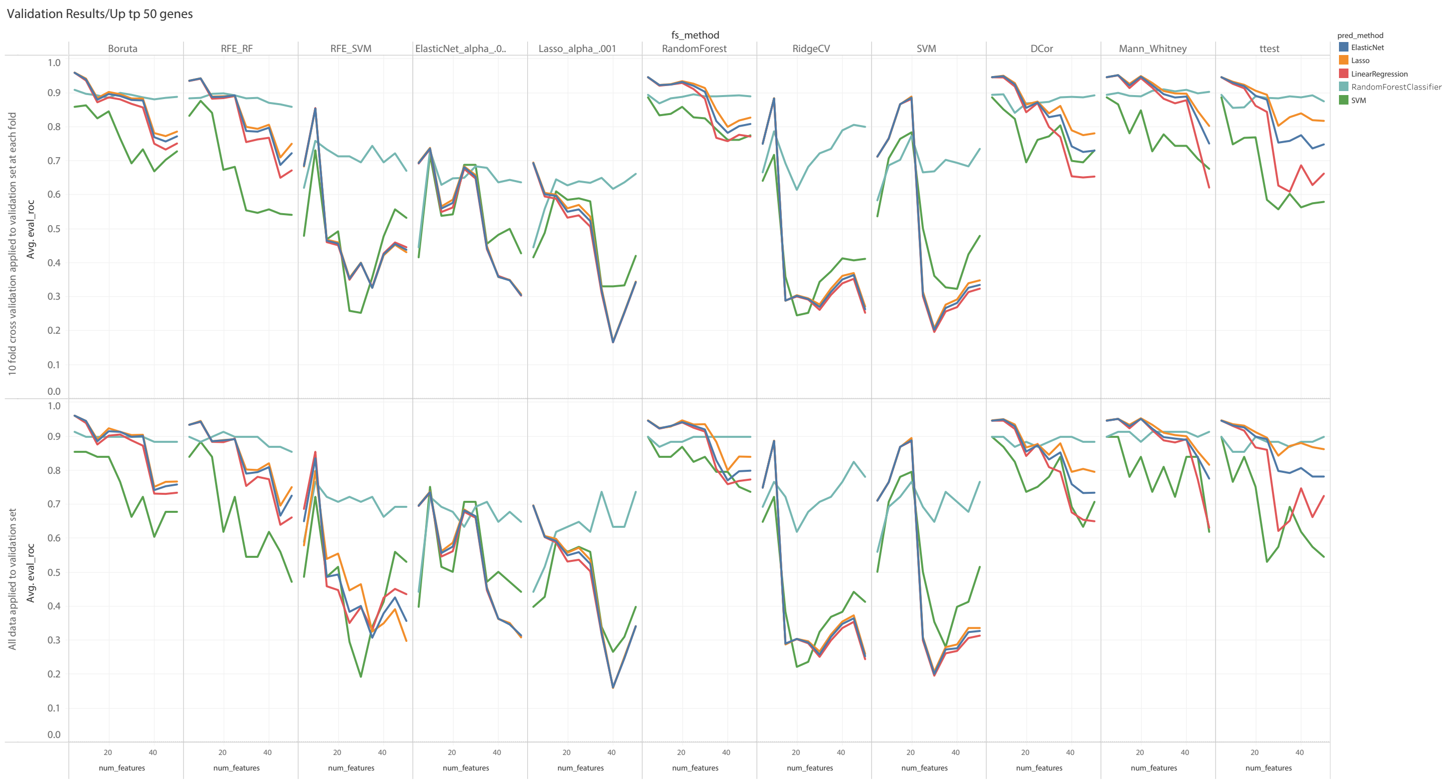

The trend of average of eval_roc for num_features broken down by fs_method vs. wix_run_id (group). Color shows details about pred_method. The data is filtered on wix_run_id, which keeps 12 of 12 members. The view is filtered on pred_method, fs_method, wix_run_id (group) and num_features. The pred_method filter keeps ElasticNet, Lasso, LinearRegression, RandomForestClassifier and SVM. The fs_method filter excludes ElasticNet_alpha_.01 and Lasso_alpha_.01. The wix_run_id (group) filter keeps 10 fold cross validation applied to validation set at each fold and All data applied to validation set. The num_features filter ranges from 5.00 to 50.00.

B

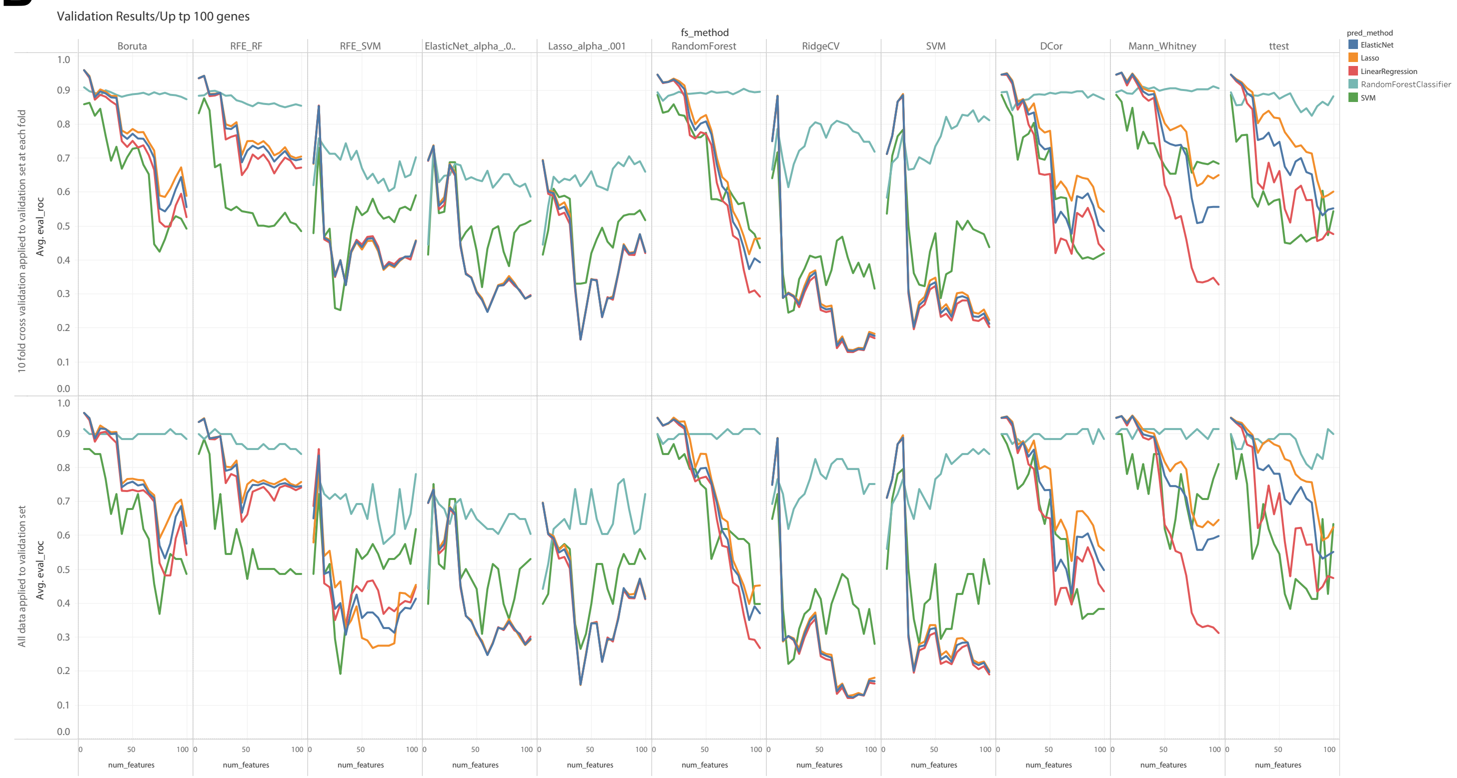

Supplement: Supplementary file 1 — Supplementary Information. [file 41598_2020_76129_MOESM1_ESM.pdf]
